# The transcription factor TAL1 and miR-17-92 create a regulatory loop in hematopoiesis

**DOI:** 10.1038/s41598-020-78629-z

**Published:** 2020-12-08

**Authors:** Annekarin Meyer, Stefanie Herkt, Heike Kunze-Schumacher, Nicole Kohrs, Julia Ringleb, Lucas Schneider, Olga N. Kuvardina, Thomas Oellerich, Björn Häupl, Andreas Krueger, Erhard Seifried, Halvard Bonig, Joern Lausen

**Affiliations:** 1 Institute for Transfusion Medicine and Immunohematology, and German Red Cross Blood Service BaWüHe, Goethe University, Sandhofstraße 1, 60528 Frankfurt, Germany; 2 Institute for Molecular Medicine, Goethe University, Theodor-Stern-Kai 7, 60590 Frankfurt, Germany; 3 Institute for Tumor Biology and Experimental Therapy, Georg-Speyer-Haus, Paul-Ehrlich-Strasse 42-44, 60596 Frankfurt am Main, Germany; 4 Department of Medicine II, Hematology/Oncology, Goethe University, Theodor-Stern-Kai 7, 60590 Frankfurt, Germany; 5 German Cancer Research Center and German Cancer Consortium, Heidelberg, Germany; 6 Frankfurt Cancer Institute, Goethe University, 60596 Frankfurt, Germany; 7 Department of Medicine, Division of Hematology, University of Washington, Seattle, WA 98195 USA; 8 Department of Eukaryotic Genetics, Institute of Industrial Genetics, University of Stuttgart, Allmandring 31, 70569 Stuttgart, Germany

**Keywords:** Cell biology, Genetics, Molecular biology, Stem cells

## Abstract

A network of gene regulatory factors such as transcription factors and microRNAs establish and maintain gene expression patterns during hematopoiesis. In this network, transcription factors regulate each other and are involved in regulatory loops with microRNAs. The microRNA cluster miR-17-92 is located within the *MIR17HG* gene and encodes six mature microRNAs. It is important for hematopoietic differentiation and plays a central role in malignant disease. However, the transcription factors downstream of miR-17-92 are largely elusive and the transcriptional regulation of miR-17-92 is not fully understood. Here we show that miR-17-92 forms a regulatory loop with the transcription factor TAL1. The miR-17-92 cluster inhibits expression of TAL1 and indirectly leads to decreased stability of the TAL1 transcriptional complex. We found that TAL1 and its heterodimerization partner E47 regulate miR-17-92 transcriptionally. Furthermore, miR-17-92 negatively influences erythroid differentiation, a process that depends on gene activation by the TAL1 complex. Our data give example of how transcription factor activity is fine-tuned during normal hematopoiesis. We postulate that disturbance of the regulatory loop between TAL1 and the miR-17-92 cluster could be an important step in cancer development and progression.

## Introduction

Hematopoiesis is a highly regulated process in which stem cells differentiate through progenitor stages to adult blood cells. Accordingly, gene expression programs are continuously adjusted to ensure cell type and lineage specific expression patterns. During differentiation transcription factors assemble large transcriptional regulatory complexes, which alter transcription directly or change expression patterns epigenetically^1,2^. This way cell type identity is established and a balance between proliferation and differentiation is ensured. Deregulated transcriptional processes are a main cause of human diseases such as cancer.

MicroRNAs are important regulators of transcription factors. These small RNA molecules bind to the 3′-UTR of protein coding target mRNAs and lead to inhibition of translation or degradation of the mRNA. MicroRNAs and transcription factors are able to create gene regulatory loops in which they regulate each other^3–5^.

miR-17-92 is encoded by the *MIR17HG* gene and is transcribed as a polycistronic transcript which yields six mature miRNAs: miR-17, miR-18, miR-19a, miR-19b, miR-20 and miR-92^6^. The microRNA cluster exhibits indispensable physiological functions in hematopoiesis and embryonic development. A mouse model with a complete miR-17-92 knockout displays postnatal lethality after birth due to hypoplastic lungs and ventricular defects. Furthermore, B-cell development is blocked at the pro- to pre-B cell transition and T-cell development is impaired^7,8^. miR-17-92 is upregulated in a number of hematopoietic malignancies and several types of solid tumors like breast, colon, lung, pancreas, prostate or stomach cancer^9–13^.

The transcription factor TAL1 is required for definitive hematopoiesis in hematopoietic stem cells^14–17^ and regulates cell type specific gene expression at the megakaryocytic/erythroid branching^18–20^. Mice exhibiting a complete TAL1 knockout are not viable and die due to a loss of hematopoiesis^14^. Furthermore, aberrant expression of TAL1 is associated with T-cell leukemia^21–23^. TAL1 is a basic-helix-loop-helix (bHLH) transcription factor, which binds regulatory DNA regions at E-boxes with the sequence CANNTG as a heterodimer with class I bHLH E-proteins like E47 and HEB^24–29^. In addition to this, TAL1 forms a core regulatory complex with cofactors like LDB1 and LMO2, which play an important role in lineage decision. TAL1 acts as an activator or repressor of gene expression in conjunction with coactivators or corepressor proteins^30–35^.

Recently, the regulation of TAL1 by microRNAs has become the subject of investigation^36–38^. TAL1 has a more than 3 kb long 3′-UTR which harbors multiple potential binding sites for microRNAs suggesting that TAL1 abundance is regulated by the activity of microRNAs. However, little is known about the microRNAs, which target TAL1 and the microRNAs, which are transcriptionally regulated by TAL1.

In this study, we demonstrate, that miR-17-92 reduces TAL1 protein amount and indirectly influences protein stability of members of the TAL1-complex. Furthermore, we show that TAL1 binds to the *MIR17HG* promoter with its heterodimerization partner E47 and down modulates transcription of the miRNA cluster. This creates a regulatory feedback loop between TAL1 and the microRNAs of the miR-17-92 cluster.

## Materials and methods

All methods were performed in accordance with the relevant guidelines and regulations. Primary human CD34^+^ cells were obtained from bone marrow of healthy volunteer donors with informed consent and approval of the ethics committee of the Frankfurt University Hospital (permit #329-10).

### Cell culture

Erythroleukemic K562 and T-cell leukemic Jurkat cells were cultured in RPMI-1640 and HEK293T cells in DMEM medium supplemented with 10% fetal calf serum, 2 mM glutamine and 1% penicillin/streptomycin. Lentiviral particles were produced in HEK293T cells as described previously^39^. K562 and Jurkat cells were transduced with TAL1 knockdown constructs targeting the following sites: shTAL1#1 5′- ATTCTTGCTGAGCTTCTTGTC-3′; shTAL1#2 5′-TCAATTCGGA ACATAGACCA-3′.

Non-specific shRNA was used as control. Control#1 5′-GACAAGAAACTAAGCAAGAAT-3′, Control#2 5′-CTTACTCTCGCCCAAGCGAGA-3′. Jurkat cells were transduced with miR-17-92, miR-17-92 mut-19a, miR-17-92 mut-19b, miR-17-92 miR-19a/b or LegoiG2 as empty vector control.

Primary human CD34^+^ cells were immunomagnetically enriched according to the manufacturer′s instructions (Miltenyi, Bergisch Gladbach, Germany). CD34^+^ cells were expanded in Stem Span (SFEMI, Stemcell Technologies, Vancouver, Canada) supplied with cytokines as described previously^34^ for 48 h. Two days after immunomagnetic isolation, CD34^+^ cells were transduced by lentiviral gene transfer with overexpression vectors and sorted according to GFP^+^ signals under sterile conditions. Subsequently, transduced CD34^+^ cells were seeded out for Colony forming assay (CFU) in methylcellulose supplied with 3% penicillin/streptomycin according to the manufacturer’s instructions (Miltenyi, Bergisch Gladbach, Germany). Colonies were counted 10–14 days after seeding.

### Gene expression analysis

Total RNA was isolated from a cell pellet of a maximum cell number of 5 × 10^6^ using the RNeasy Mini Kit (Qiagen, Hilden, Germany). Complementary DNA was subsequently generated by using Omniscript reverse transcriptase (Qiagen). Quantitative reverse transcriptase PCR (qRT-PCR) was performed using SYBR Green PCR Mastermix (Eurogentec, Luettich, Belgium). Values obtained by qRT-PCR were normalized against glyceraldehyde-3-phosphate dehydrogenase (GAPDH) expression values. Primer sequences are given (Supplementary Material S1). Expression levels of mature members of the miR-17-92 cluster were analysed using the “Real-Time PCR Assay Kit” (Signosis).

### Mass spectrometry

Jurkat cells overexpressing miR-17-92 were cultivated in heavy SILAC (stable isotope labeling by amino acids in cell culture) medium and control cells transduced with the empty vector control (LegoiG2) were cultivated in light SILAC medium for five passages (14 days). Similar cell numbers were harvested and nuclear lysates were prepared. 20 µg of each sample were combined in one 1.5 ml reaction tube together with 4 × NuPage buffer and 10 × sample reducing agent to a total volume of 50 µl. Samples were analyzed by mass spectrometry. Values were normalized to the control sample by calculating the heavy/light (H/L)-ratio. Bioinformatic analysis included all proteins downregulated by a (H/L)-ratio of at least 0.7 compared to the control sample. Data are available via ProteomeXchange with identifier PXD018052.

### Chromatin immunoprecipitation

ChIP-assays were performed according to the Abcam-X-ChIP protocol with modifications as previously described^34^. 3–10 µg of specific antibodies were used (Supplementary Material S1). For quantitative PCR 1 µl of eluted DNA was used (Primer pairs are listed in Supplementary Material S1).

### Luciferase assay

The full length 3′UTR of TAL1 and three deletion constructs were cloned behind a luciferase reporter gene with promoter (pMirTarget). Binding sites of the represented miRNAs were mutated by site directed mutagenesis. Transfection of K562 cells, cell lysis, luciferase measurement and value normalization and analysis were performed as described previously^40^. HEK293T cells were transfected and lysed as described previously^39^. β-galactosidase activity for normalization was measured 5 min after addition of buffer (11.1 mM MgCl2, 50 mM β-Mercaptoethanol, 3.25 mM o-Nitrophenyl-β-d-galactopyranosid (ONPG), 74.4 mM sodium phosphate) at an absorption of 420 nm.

### Western blotting

Western blotting was performed according to standard techniques. Protein transfer was done using a semi-dry system (Biorad). Western blots were analyzed and quantified with the Odyssey system (primary and secondary antibodies listed in Supplementary Material S1). Tubulin or Histone 3 expression was assessed as a loading control. Uncropped images are given (Supplementary Fig. S10–S14).

### Statistics

Experiments were performed at least three times and were statistically analyzed using GraphPad Prism software. The error bars represent the standard deviation from the mean. P values were calculated using the Student’s t-test from at least three determinations. P values < 0.05 were considered statistically significant (*P < 0.05; **P < 0.01; ***P < 0.001).

## Results

### Identification of downstream targets of miR-17-92

To identify targets of miR-17-92 at the protein level, we combined overexpression of miR-17-92 with SILAC based mass spectrometry. Because of the involvement of miR-17-92 in T-cell development we used Jurkat cells, an immortalized cell line of human T-lymphocytes.

**Figure 1:**
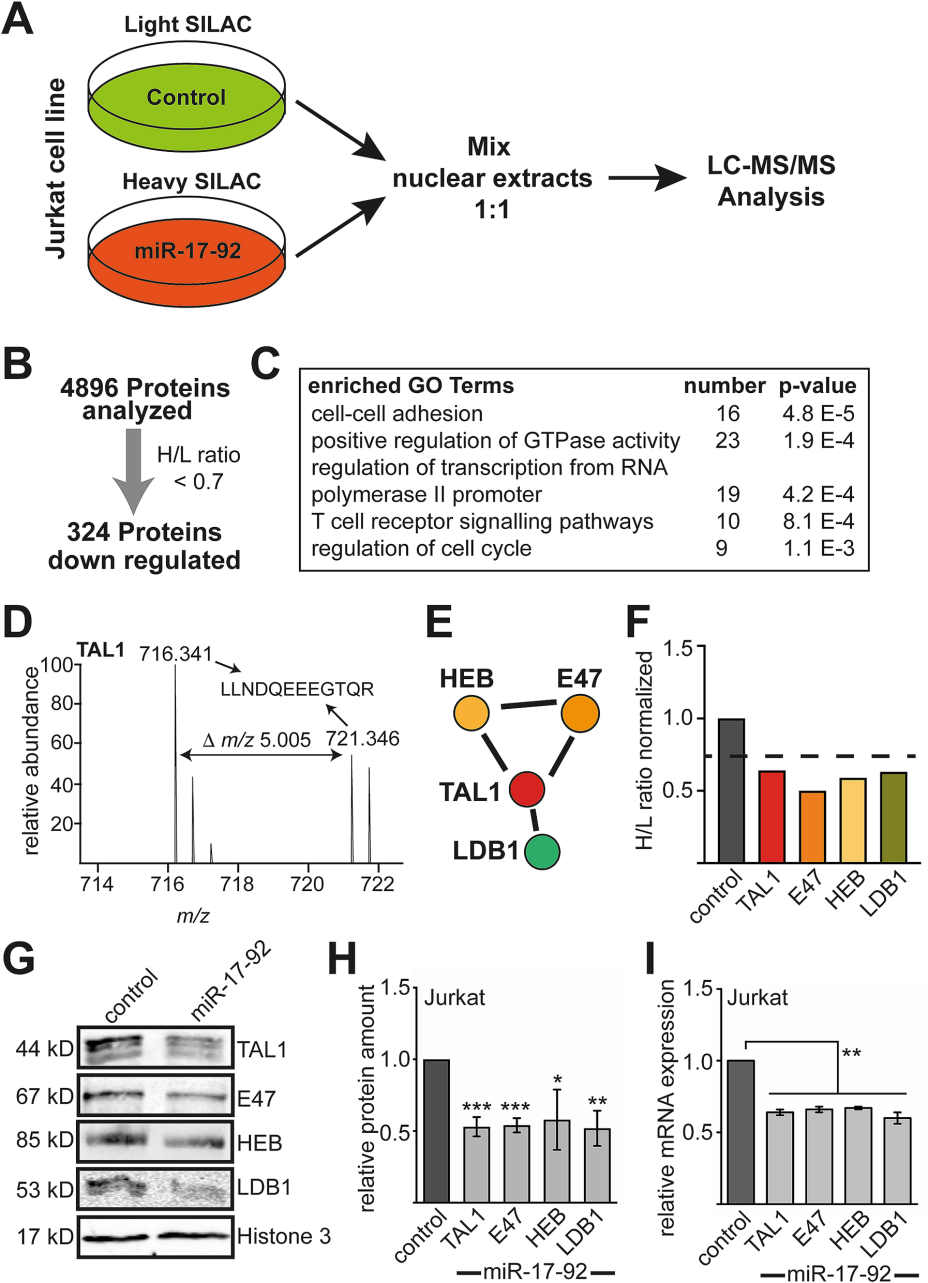
SILAC based mass spectrometry reveals miR-17-92 targets. (**A**) Jurkat cells were transduced with miR-17-92 and the corresponding empty vector control LegoiG2. The control cells were cultured for five passages in light SILAC medium, containing normal amino acids. miR-17-92 overexpressing cells were cultured in heavy SILAC medium labelled with heavy isotopes. Nuclear extracts were prepared and light and heavy SILAC labelled cells were mixed in a one to one ratio and applied to LC–MS/MS analysis. (**B**) 4896 proteins were analysed, thereof 324 proteins were downregulated by an H/L ratio of at least 0.7. (**C**) GO-term analysis of downregulated proteins using DAVID. (**D**) MS-spectrum of a TAL1 peptide shows downregulation of TAL1 protein in the miR-17-92 sample. (**E**) Protein interaction network analysis of the 324 downregulated proteins using STRING revealed that the TAL1 interactors HEB, E47 and LDB1 are among the downregulated proteins. (**F**) The members of the TAL1 interaction network E47, HEB and LDB1 were downregulated by an H/L ratio of at least 0.7. (**G**) miR-17-92 was overexpressed in Jurkat cells under non-SILAC conditions. Protein levels of TAL1, E47, HEB and LDB1 were analysed using western blot with the corresponding antibodies. (**H**) Quantification of the protein amount of TAL1, E47, HEB and LDB1 normalized to a western blot against histone 3. The quantification from western blot in the control cells was set as one. Data are given as relative protein amount compared to the control transduced with empty vector. Western blots were performed at least three times. (**I**) mRNA expression of TAL1, E47, HEB and LDB1 was reduced upon miR-17-92 overexpression. Data are given as relative mRNA amount compared to the control transduced with empty vector. The p-values were calculated using Student’s t-test from at least three independent determinations. *P < 0.05; **P < 0.01; ***P < 0.001.

We moderately overexpressed miR-17-92 in Jurkat cells by transduction resulting in ten times higher miR-17-92 levels than in control cells (Fig. S1). miR-17-92 positive cells were grown in heavy isotope labeled SILAC medium and control cells in light SILAC medium for five passages. Nuclear extracts were mixed in a one to one ratio and subjected to liquid-chromatography mass-spectrometry/mass-spectrometry (LC–MS/MS) (Fig. 1A, Fig. S1). A total number of 4896 proteins was measured, 324 of which were downregulated in the miR-17-92 sample with at least an H/L ratio < 0.7 (Fig. 1B and Table S1). These proteins constitute potential direct and indirect miR-17-92 targets.

Subsequently, we performed gene ontology (GO)-term analysis using DAVID^41^ and found that the GO-terms cell–cell adhesion, regulation of GTPase activity, regulation of transcription, T-cell receptor signaling pathways and regulation of cell cycle were enriched (Fig. 1C). To further analyze the functional relationship between the 324 identified potential miR-17-92 targets, we examined if they form interaction networks by using STRING analysis^42^.

We found that the basic helix-loop-helix transcription factor TAL1 was reduced in miR-17-92 expressing Jurkat cells. The MS-spectrum of a TAL1 peptide is shown (Fig. 1D). Furthermore, the TAL1 interaction partners HEB (TCF12), E47 (TCF3) and LDB1 were reduced (Fig. 1E,F). These proteins are known to create a gene regulatory complex on TAL1 target genes in hematopoietic cells^43^.

Our data indicated that miR-17-92 is a regulatory microRNA of the TAL1 transcriptional complex. To verify these results, we overexpressed miR-17-92 under non-SILAC conditions. In agreement with our SILAC data, we detected reduction of TAL1, E47, HEB and LDB1 on the protein level upon miR-17-92 overexpression (Fig. 1G,H). These transcription factors were reduced at the mRNA level upon miR-17-92 overexpression (Fig. 1I). The down-regulation of the TAL1 transcriptional complex upon miR-17-92 expression should lead to altered expression of TAL1 target genes. Within the 324 down-regulated proteins upon miR-17-92 overexpression some alterations are likely caused indirectly by the loss of TAL1 function. Indeed, comparison of TAL1 target genes, using the integrated ChEA-ChIP-database^44^, showed that within the 324 miR-17-92 targets 97 genes are direct target genes of TAL1 (Fig. S2). Some of these genes have been described in Jurkat cells, such as CD4 and NKX 3.1^24,29,45,46^.

### TAL1 is a target of miR-17-92

**Figure 2:**
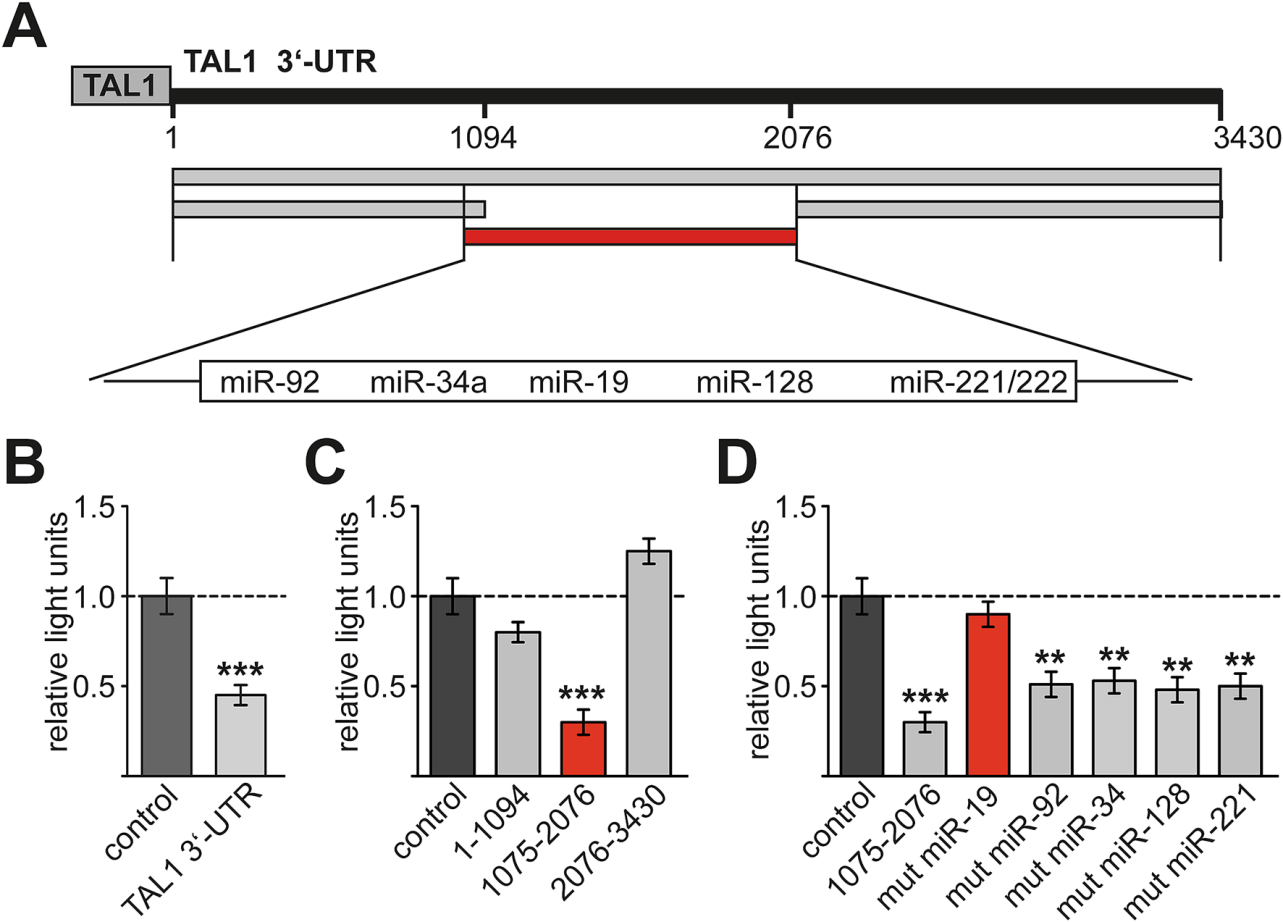
A miR-19 binding site in the 3′-UTR of TAL1 mediates repression. (**A**) Outline of the 3430 bp long 3′-UTR of TAL1. Using TargetScan several miRNA binding sites within the 3′-UTR of TAL1 were identified^74^. MiRNA binding sites are shown, among others miR-19 and miR-92 of the miR-17-92 cluster. The whole length 3′-UTR and three deletion constructs from position 1–1094, 1075–2076 and 2076–3430 were cloned into pMirTarget behind a luciferase reporter gene. (**B**) Luciferase reporter gene assay was performed in K562 cells. The full-length construct of the TAL1 3′-UTR exhibited lower luciferase activity compared to the empty vector control. Data are shown as relative light units gathered from at least three independent transfections. Values gathered with empty vector were set as one. (**C**) Luciferase reporter assay with three deletion constructs of the TAL1 3′-UTR. The middle fragment from position 1075–2076 (red bar) displayed a reduced luciferase activity. The other tested fragments did not alter the luciferase activity. Data are shown as relative light units gathered from at least three independent transfections. Values gathered with empty vector were set as one. (**D**) The microRNA binding sites in the middle part of the 3′-UTR were mutated. The mutation of the binding site of miR-19 led to a loss of the repressive effect. Data are shown as relative light units gathered from at least three independent transfections. Values gathered with empty vector were set as one. The p-values were calculated using Student’s t-test from at least three independent determinations. **P < 0.01; ***P < 0.001.

We identified binding sites for microRNAs of the miR-17-92 cluster within the 3′-UTR of TAL1, but not in the 3′-UTR of HEB, E47 and LDB1 (Fig. S3). Subsequently, we analyzed the 3′-UTR of TAL1 in a reporter gene assay (Fig. 2A). These experiments were performed in K562 cells, which express TAL1 and could be better transfected compared to Jurkat cells. Furthermore, the TAL1 complex with HEB, E47 and LDB1 is known to be functional in K562 cells^43^.

The full-length 3′-UTR reporter constructs led to a 50% lower activity of the luciferase reporter compared to the empty reporter vector (Fig. 2B). Subsequently, we divided the 3′-UTR into three deletion constructs of similar size, 1–1094, 1075–2076 and 2076–3401 (Fig. 2A). The construct from 1075–2076 repressed the luciferase reporter significantly, whereas the other regions displayed no influence in this setting (Fig. 2C). This indicated that the central region of the TAL1 3′-UTR harbors binding sites for microRNAs, which lead to repression. Interestingly, microRNA binding sites for miR-19 and miR-92 of the miR-17-92 cluster are located within this region of the 3′-UTR of TAL1. Furthermore, potential binding sites for miR-34, miR-128 and miR-221/222 reside within this region (Fig. 2A, Fig. S3).

To examine if these sites play a role in repression, we mutated the microRNA binding sites within the TAL1 3′-UTR 1075–2076 reporter construct. Mutation of each site attenuated repression, however mutation of the miR-19 binding site strongly relieved repression of the 1075–2076 construct (Fig. 2D). These results indicate that the region of 1075–2076 of the TAL1 3′-UTR is indeed targeted by microRNAs and that miR-19 might be involved.

### MiR-19 of the miR-17-92 cluster is implicated in TAL1 repression

**Figure 3:**
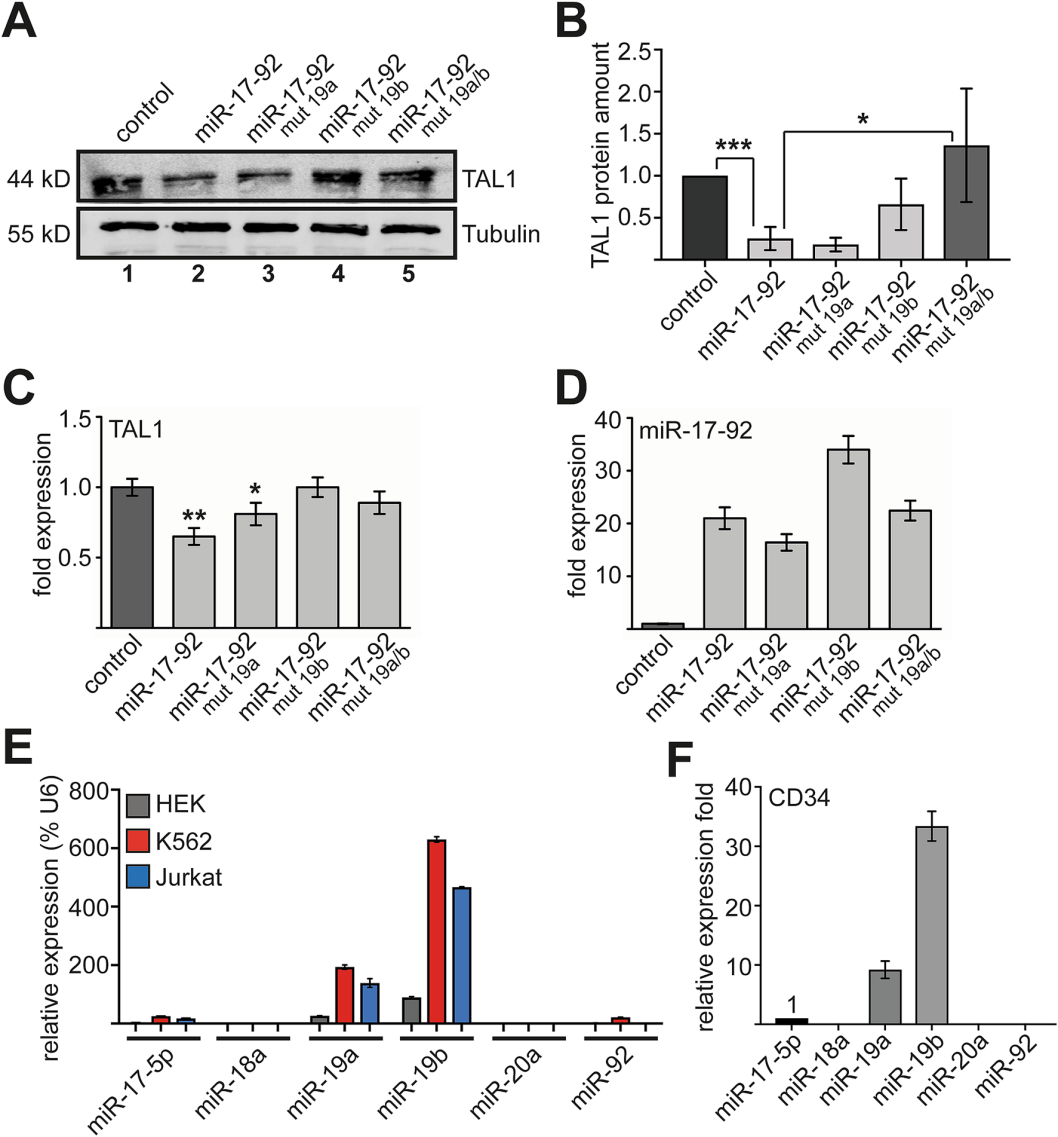
The repressive effect of miR-17-92 on TAL1 is lost by mutation of the miR-19 seed sequences. (**A**) Western blot showing the effect of miR-17-92 wild type and mutants on TAL1 protein amount. TAL1 protein amount was reduced upon transduction of miR-17-92 compared to transduction with control vector in Jurkat cells (compare lane 1 to lane 2). The seed sequences of miR-19a and miR-19b of the miR-17-92 cluster were mutated, respectively. Western blot analysis showed that the inhibiting effect of miR-17-92 on TAL1 was lost after mutation of miR-19b and a double mutation of miR-19a/b (lane 4 and lane 5). (**B**) Quantification of TAL1 protein levels by western blot analysis using the Odyssey system. Relative protein amounts are shown compared to values gathered upon transduction with empty vector. (**C**) Quantification of TAL1 mRNA levels by qRT-PCR upon transduction of miR-17-92 wild type and mutants compared to transduction with control vector in Jurkat cells. Data were normalized to expression of GAPDH and values are displayed as fold expression compared to the empty vector control. Evaluation was performed three times. (**D**) Overexpression levels of miR-17-92 wild type and mutant constructs were determined by qRT-PCR. Primers against unprocessed miR-17-92 were used. Values were normalized to expression levels of GAPDH and are shown as fold expression compared to cells transduced with empty vector. (**E**) Expression levels of the six mature members of the miR-17-92 cluster were analysed using the “Real-Time PCR Assay Kit” (Signosis). Mature miR-19a and miR-19b show the highest expression in HEK, K562 and Jurkat cells. Data are shown in percent relative to values gathered for RNUB6 (U6). (**F**) Expression levels of the six mature members of the miR-17-92 cluster in CD34^+^ cells were analysed using the “Real-Time PCR Assay Kit” (Signosis). CD34^+^ cells show high expression of miR-19a and the even higher expression of miR-19b. Values are shown as fold expression compared to miR-17-5p expression, which was set as one. Error bars were calculated using standard deviation from at least three independent values. The p-values were calculated using Student’s t-test from at least three independent measurements. *P < 0.05; **P < 0.01.

Analysis of the 3′-UTR of TAL1 suggested that miR-17-92 represses TAL1 expression. In line with this notion, increased miR-17-92 expression reduced TAL1 at the protein level in Jurkat cells (Fig. 3A, compare lane 1 and 2). Mutation of the seed sequence of miR-19a, within the miR-17-92 construct did not affect this reduction (Fig. 3A, lane 3, see Fig. S4 for sequences). However, mutation of the seed sequence of miR-19b and the double mutant miR-19a/b diminished the repressive effect of miR-17-92 on TAL1 protein (Fig. 3A,B, lane 4 and lane 5). Figure 3B shows the quantification of the western blot.

The reduction of TAL1 was mainly detected on the protein level as TAL1 mRNA was not reduced to the same extent by miR-17-92. This moderate effect was diminished with the miR-19b mutant and the miR-19a/b double mutant (Fig. 3C). These results were not caused by different expression levels, as the miR-17-92 constructs were overexpressed to a similar degree (Fig. 3D). Collectively, these data strongly suggest that miR-19 of the miR-17-92 cluster targets TAL1 in Jurkat cells. Similarly, overexpression of miR-19 in K562 cells decreased TAL1 protein amount (Fig. S5).

MiR-19a and miR-19b target the same sequence and should be partly functionally redundant. However, mutation of miR-19b within the miR-17-92 cluster had a greater effect on TAL1 targeting in our experiment (Fig. 3A,B). This discrepancy could partly be explained by different abundance of the two variants. Indeed, we detected more endogenous mature miR-19b transcript than miR-19a transcript in Jurkat and K562 cells, HEK293 cells were included as a non-hematopoietic control (Fig. 3E). Also note that miR-19a and miR-19b are the most prominently expressed mature microRNAs of miR-17-92 in K562 and Jurkat cells (Fig. 3E) and also in primary human CD34^+^ progenitor cells (Fig. 3F).

### Loss of TAL1 stimulates E-protein degradation

**Figure 4:**
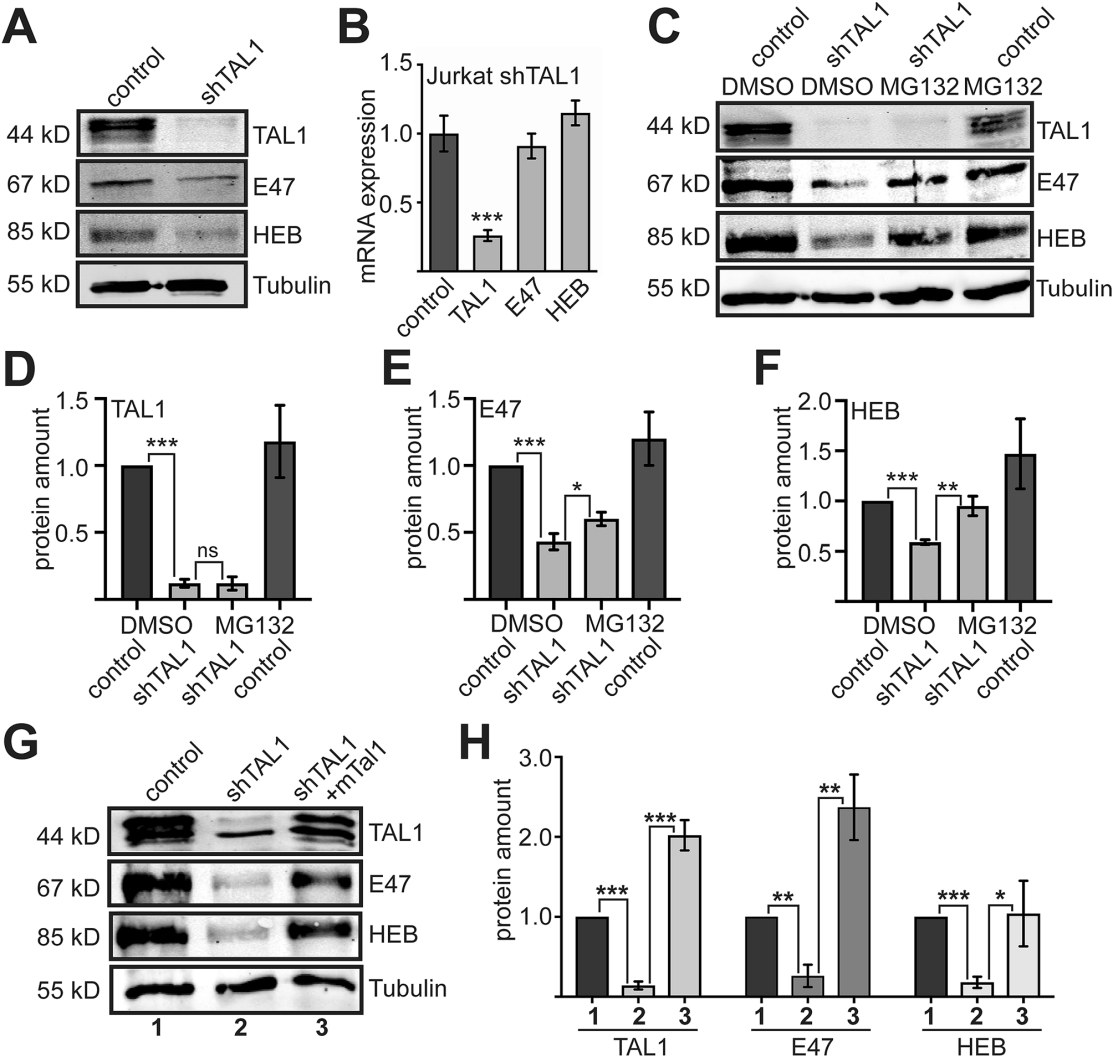
TAL1 knockdown destabilizes E-Proteins. (**A**) Western blot analysis of TAL1, E47 and HEB upon TAL1 knockdown. Jurkat cells were transduced with a small hairpin vector against TAL1 (shTAL1) or control vector with a non-targeting shRNA (control). Western blot with antibodies against TAL1, E47 and HEB revealed reduced protein amount upon knockdown of TAL1. Western blot against Tubulin served as loading control. (**B**) Analysis of the mRNA level upon knockdown of TAL1 by qRT-PCR with primer pairs recognizing the given mRNAs. TAL1 is decreased in TAL1 knockdown cells, while HEB and E47 mRNA level remained unchanged. Values were normalized with values gathered for GAPDH. (**C**) Treatment of TAL1 knockdown Jurkat cells with the protease inhibitor MG132 restored E47 and HEB expression. TAL1 knockdown cells were treated with 3 µM of the proteasome inhibitor MG132 for eight hours. Cells were harvested and applied to western blot analysis against TAL1, E47 and HEB. Western blot against Tubulin served as control. (**D**) Measurement of TAL1 protein amount upon TAL1 knockdown by western blot and quantification using the Odyssey-System. Values were normalized against the loading control Tubulin and are displayed as relative protein amount compared to western blot from cells, which were transduced with a control shRNA. (**E**) Measurement of E47 protein amount upon TAL1 knockdown by western blot and quantification using the Odyssey-System. Values were normalized against the loading control Tubulin and are displayed as relative protein amount compared to western blot from cells, which were transduced with a control shRNA. (**F**) Measurement of HEB protein amount upon TAL1 knockdown by western blot and quantification using the Odyssey-System. Values were normalized against the loading control Tubulin and are displayed as relative protein amount compared to western blot from cells, which were transduced with a control shRNA. (**G**) Rescue of TAL1 expression in TAL1 knockdown Jurkat cells restores E47 and HEB protein expression. TAL1 knockdown cells were additionally transduced with murine Tal1 (mTal1) to restore TAL1 protein amount. Western blot analysis revealed a rescue of E47 and HEB protein amount. Tubulin served as loading control. (**H**) Measurement of TAL1, E47 and HEB protein amount upon TAL1 knockdown and restoration of TAL1 expression by western blot and quantification using the Odyssey-System. Quantification of TAL1, E47 and HEB protein level upon re-expression of TAL1 expression levels in shTAL1 Jurkat cells with mTal1. Values were normalized against the loading control Tubulin and are displayed as relative protein amount compared to western blot from cells, which were transduced with a control shRNA. Error bars were determined from at least three independent experiments. P-values were calculated using Student’s t-test from at least three independent western blots. One representative blot is shown. *P < 0.05; **P < 0.01; ***P < 0.001.

Our analysis indicated that TAL1 and its heterodimerization partners E47 and HEB were reduced upon increased miR-17-92 expression (compare Fig. 1D and G). Furthermore, we established evidence that miR-17-92 directly diminishes TAL1. Subsequently, we examined, if loss of TAL1 upon miR-17-92 expression was causally connected to reduced E47 and HEB expression. Knockdown of TAL1 diminished E47 and HEB expression on the protein level (Fig. 4A), but not at mRNA level (Fig. 4B). This indicates that loss of TAL1 reduces E47 and HEB post-transcriptionally. It has been demonstrated in other cases, that heterodimerization of E-proteins can influence protein stability^26^. It seemed reasonable, that loss of TAL1 leads to increased protein degradation of E47 and HEB. Because heterodimerization plays a central role on target gene selection and transcriptional activity we focused on E47 for further analysis of this notion. Subsequently, we treated TAL1 knockdown cells with the proteasome inhibitor MG132. TAL1 protein amount was unchanged by MG132 treatment. Interestingly, E47 and HEB protein amount increased after treatment with MG132 in shTAL1 cells (Fig. 4C). TAL1 protein levels were not influenced by MG132 (Fig. 4D). Expression of E47 and HEB increased to control levels upon treatment with MG132 (Fig. 4E,F). We conclude, that the effect of TAL1 knockdown was reversed by inhibition of proteasomal degradation. This also suggests, that reduction of TAL1 within the cells increased degradation of E47 and HEB. Note that at the mRNA level E47 and HEB were not decreased (Fig. S6).

To verify the notion that E47 and HEB protein abundance is dependent on the presence of TAL1, we performed a rescue experiment by introducing murine Tal1 (mTal1) into shTAL1 cells. Murine TAL1 reestablished TAL1 expression because it is not affected by the used shRNA against human TAL1. This reintroduction of TAL1 rescued expression of E47 and HEB at the protein level (Fig. 4G,H). The mRNA amount of E47 and HEB remained unchanged (Fig. S6).

### TAL1 binds to the promoter of the miR-17-92 host gene ***MIR17HG***

**Figure 5:**
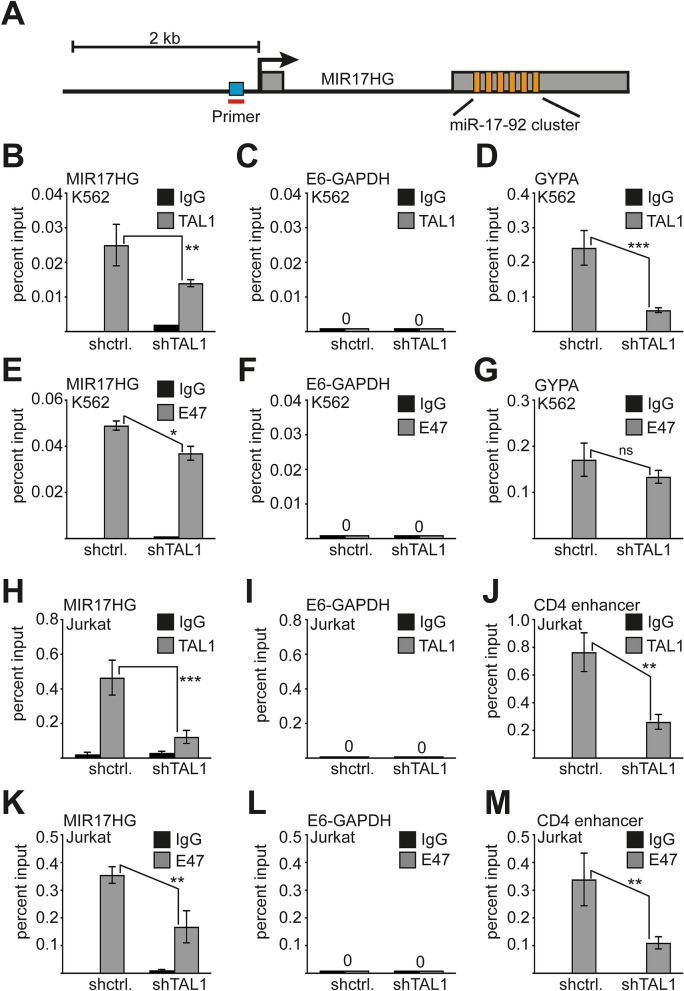
TAL1 and E47 bind to the *MIR17HG* promoter in chromatin immunoprecipitation (ChIP) assay. (A) Outline of the *MIR17HG* gene with the promoter region, a noncoding exon (small grey box), the intron region (shown as black line) and the second exon (big grey box), including the miR-17-92 cluster. The coding areas for the six individual miRNAs are shown in orange. An E-box is located in front of the transcription start site (TSS) (blue box). (**B**) ChIP-assay in K562 control cells (control) and TAL1 knockdown cells (shTAL1). TAL1 binds close to the TSS of *MIR17HG*, this binding is reduced upon knockdown of TAL1. (**C**) TAL1 did not bind to a negative control region of Exon-6-GAPDH (E6-GAPDH). (**D**) TAL1 binding was detected at the GYPA promoter in K562 cells. GYPA is a known target gene of TAL1 in K562 cells. TAL1 binding to GYPA was reduced upon knockdown of TAL1. (**E**) ChIP assay against E47 in K562 control cells (control) and TAL1 knockdown cells (shTAL1). E47 binds close to the TSS of *MIR17HG*, this binding is reduced upon knockdown of TAL1. (**F**) E47 did not bind to a negative control region of Exon-6-GAPDH (E6-GAPDH) in K562 cells. (**G**) E47 binds at the GYPA promoter in K562 cells. GYPA is a known target gene of TAL1 and E47 in K562 cells. E47 binding to GYPA was not significantly reduced upon knockdown of TAL1. (**H**) TAL1 binding at the *MIR17HG* promoter in Jurkat cells. TAL1 binding to the *MIR17HG* promoter was reduced upon TAL1 knockdown. (**I**) No TAL1 binding was shown at E6-GAPDH in Jurkat cells. (**J**) TAL1 was detected at the CD4 enhancer in Jurkat cells. CD4 is a known TAL1 target gene in Jurkat cells. Knockdown of TAL1 reduced TAL1 binding. (**K**) ChIP assay with an antibody against E47 in Jurkat cells. E47 bound to the *MIR17HG* promoter. This binding was reduced upon TAL1 knockdown. (**L**) No binding was found at Exon 6 GAPDH. (**M**) The CD4 enhancer displayed E47 binding, which was reduced upon TAL1 knockdown. All experiments were performed at least three times with independent ChIP lysates. Quantitative PCR was performed with specific primers and SYBR green. Error bars were determined from at least three independent experiments. P-values were calculated using Student’s t-test from at least three experiments. **P < 0.01; ***P < 0.001.

Transcription factors and microRNAs can be engaged in regulatory loops. Thus, we examined the possibility that TAL1 regulates expression of miR-17-92. The *MIR17HG* gene is located on chromosome 13 and the miR-17-92 cluster resides within the second non-protein-coding exon. It encodes a pri-microRNA with six microRNAs (Fig. 5A). There is an E-box located in the promoter region of *MIR17HG*^47^ (Fig. 5A, blue box).

We performed chromatin immunoprecipitation (ChIP) in K562 cells and located TAL1 binding close to the first exon of *MIR17HG* (Fig. 5B), but not to a control region (Fig. 5C). Binding of TAL1 to the promoter of *glycophorin A* (*GYPA*) served as positive control (Fig. 5D). Reduced binding of TAL1 to the promoters was measured upon TAL1 knockdown (Fig. 5B,D). Because TAL1 and its heterodimerization partner E47 are functionally connected, we also examined E47 binding upon TAL1 knockdown at *MIR17HG* and the known TAL1 target gene *GYPA* in K562 cells (Fig. 5E–G). Knockdown of TAL1 reduced E47 binding to *MIR17HG* (Fig. 5E). However, there was no significant effect on E47 binding upon TAL1 knockdown to the *GYPA* promoter in K562 cells (Fig. 5G).

Furthermore, we detected binding of TAL1 to the promoter region of *MIR17HG* in Jurkat cells (Fig. 5H). No binding was found at a control region (Fig. 5I). TAL1 bound to the CD4 gene enhancer, a known TAL1 target^24^ (Fig. 5J). Knockdown of TAL1 led to reduced binding of TAL1 to its targets in Jurkat cells (Fig. 5H–J). E47 also bound to the *MIR17HG* promoter and this binding was reduced upon TAL1 knockdown (Fig. 5K). No E47 binding was detected at a negative control region (Fig. 5L), but E47 was found at the CD4 gene enhancer (Fig. 5M). This binding was reduced upon loss of TAL1.

These data established *MIR17HG* as a TAL1/E47 target gene and show that TAL1 and E47 are functionally related at the *MIR17HG* promoter. ChIP against the other identified partner of TAL1, HEB, failed due to antibody limitations. Our data show that the regulation of *MIR17HG* by TAL1 is relevant in distinct lineages as K562 and Jurkat cells.

### TAL1 and E47 regulate expression of *MIR17HG*

Our data support the idea that regulation of *MIR17HG* by TAL1 is a general mechanism, as TAL1 is located on the *MIR17HG* promoter in K562 and Jurkat cells.

**Figure 6:**
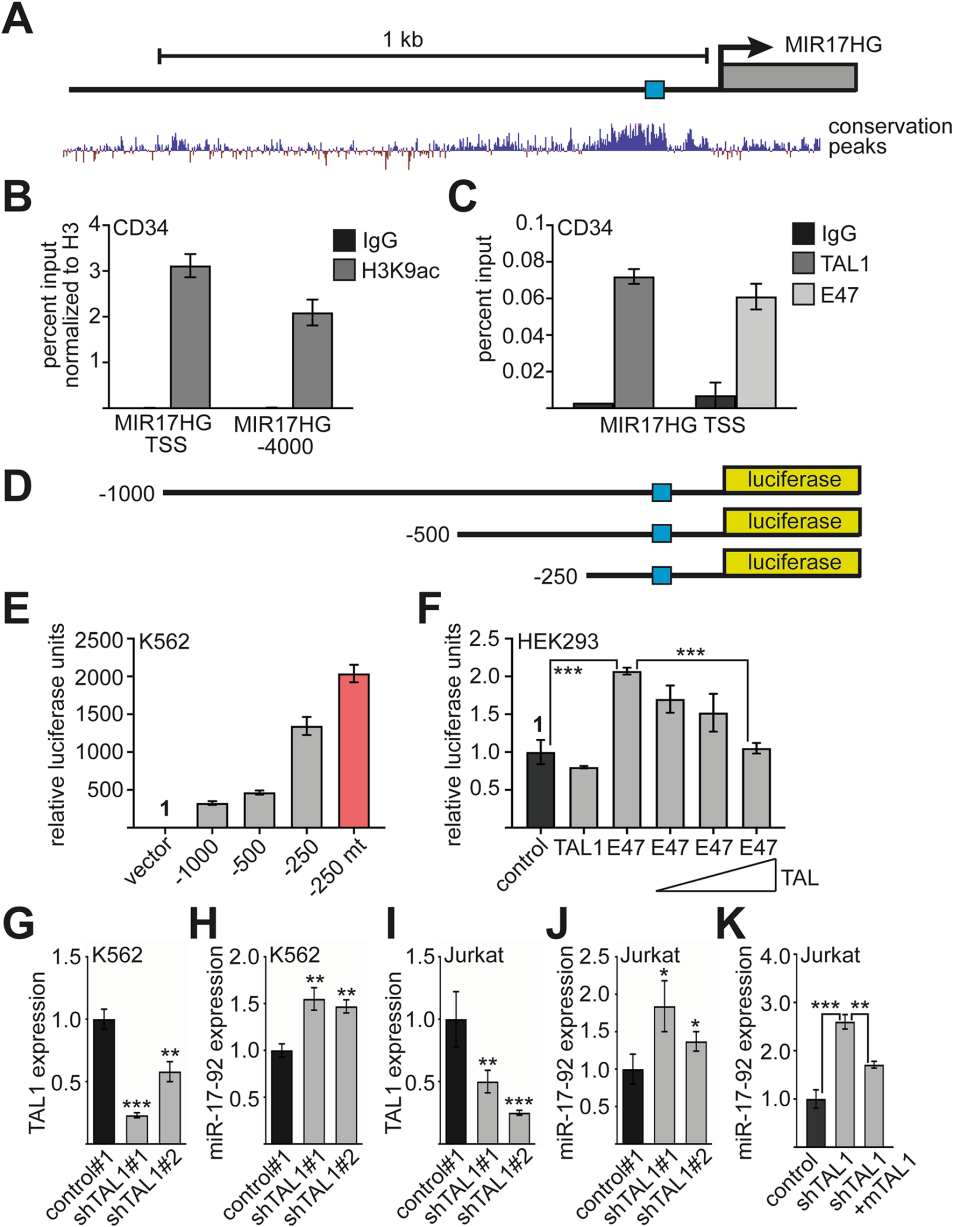
*MIR17HG* expression is regulated by TAL1/E47. (**A**) Schematic illustration of the *MIR17HG* locus with a focus on the 5′-region. Shown is the promoter region and the MYC-site close to the transcriptional start site (TSS, blue box). The lower part displays a screen shot of the human genome browser presentation of conserved regions. (**B**) Analysis of H3K9ac of the *MIR17HG* promoter in primary human CD34^+^ cells. ChIP-assay was performed with an anti-H3K9ac antibody. Primers were located around the E-box (*MIR17HG* TSS) and at a region -4000 of the TSS. (**C**) TAL1 and E47 binding to the *MIR17HG* promoter in primary human CD34^+^ cells. ChIP-assay was performed with an anti-TAL1 and anti-E47 antibody. Primers were located around the E-box (*MIR17HG* TSS) and at a region -4000 of the TSS. Error bars give the standard deviation from at three independent determinations. (**D**) Luciferase constructs of 1000, 500 and 250 bp in length were prepared and cloned into pGL4.10 in front of a luciferase reporter gene. The blue box displays the E-box. (**E**) In a luciferase assay, the promoter activity of the three different constructs was measured after transfection into K562 cells. All three constructs show promoter activity. The first 250 base pairs show the highest activity. A mutation of the E-box led to increased promoter activity. Values are given as relative luciferase activity. Activity of the empty luciferase vector pGL4.10 was set as one. (**F**) Cotransfection of TAL1 and E47 with the 500 bp construct in HEK293T cells. Increasing TAL1 amount decreased the activating effect of E47 on the *MIR17HG* promoter. As control the 500 bp construct was set as one. (**G**–**J**) Expression of miR-17-92 in K562 and Jurkat cells after TAL1 knockdown. Shown is the TAL1 and miR-17-92 mRNA Expression upon transduction with two distinct shRNAs. qRT-PCR values were normalized against GAPDH expression and are displayed as relative expression compared to the control transduction. (**G**) TAL1 expression upon knockdown with two distinct TAL1 shRNAs in K562 cells. (**H**) Expression of miR-17-92 in K562 cells after TAL1 knockdown. (**I**) TAL1 expression upon knockdown with two distinct TAL1 shRNAs in Jurkat cells. (**J**) Expression of miR-17-92 in Jurkat cells after TAL1 knockdown. (**K**) Expression of miR-17-92 upon TAL1 knockdown and restoration of TAL1 expression with murine TAL1 (mTal1). Error bars were determined from at least three independent experiments. P-values were calculated using Student’s t-test from at least three experiments. **P < 0.01; ***P < 0.001.

The 5′-region of *MIR17HG* has a conserved region close to the transcriptional start site (Fig. 6A). Within this conserved region an E-box with the sequence CACATG is located (Fig. S7). This sequence is also present in mouse and zebrafish (not shown). We found that the promotor of *MIR17HG* is acetylated at H3K9 in CD34^+^ cells, which indicates its transcriptional activity (Fig. 6B). Subsequently, we examined if TAL1 and E47 are also bound to *MIR17HG* in primary human CD34^+^ progenitor cells. Indeed, TAL1 and E47 were detected at the *MIR17HG* promoter in CD34^+^ cells (Fig. 6C).

To further examine TAL1/E47 activity on the promoter, we established three *MIR17HG* promoter luciferase reporter gene constructs (Fig. 6D). All four promoter constructs displayed robust promoter activity in K562 cells, however the -250 construct had the highest activity (Fig. 6E). Interestingly mutation of the E-box from CACTAG to CACAAG led to increased promoter activity in K562 cells (Fig. 6E).

Subsequently, we cotransfected TAL1 and E47 with the *MIR17HG* promoter reporter gene, respectively. This experiment was performed in HEK293 cells, because they do not express endogenous TAL1. TAL1 slightly reduced the activity of the *MIR17HG* promoter reporter (Fig. 6F). E47 activated the promoter construct and cotransfection of rising amounts of TAL1 led to decreased promoter activity (Fig. 6F). This result is in line with reports that the TAL1/E47 heterodimer is less activating than the E47 homodimer^24,25^.

To examine the notion that TAL1 acts repressive on miR-17-92 expression, we knocked down TAL1 in K562 cells (Fig. 6G). This resulted in an increase of miR-17-92 expression in K562 cells (Fig. 6H). Similarly, we knocked down TAL1 in Jurkat cells (Fig. 6I), which led to increased miR-17-92 expression (Fig. 6J). Subsequently, we found that the increased miR-17-92 level upon TAL1 knockdown was partly rescued by re-expression of TAL1 (Fig. 6K).

In summary, our results show the TAL1/E47 complex regulates *MIR17HG* and that TAL1 represses miR-17-92 expression in K562 and Jurkat cells.

### Influence of miR-17-92 on differentiation

**Figure 7:**
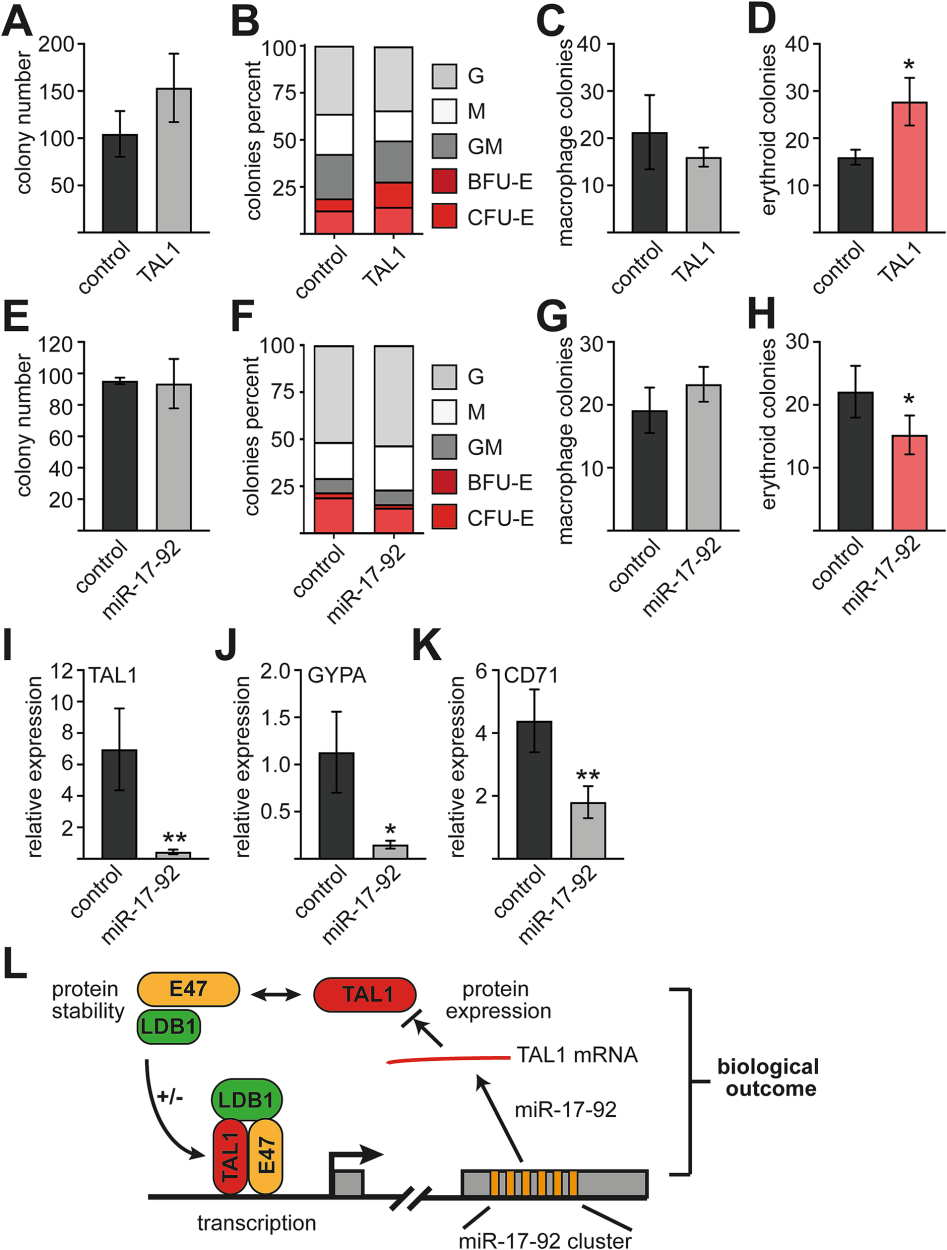
TAL1 and miR-17-92 overexpression in CD34^+^ cells influences erythroid differentiation in a CFU assay. (**A**–**D**) Influence of TAL1 on differentiation. CD34^+^ cells were transduced with TAL1 and the empty vector LegoiG2 (control), respectively. GFP^+^ cells were plated out on methylcellulose and colonies were counted 10–14 days later. (**A**) Total colony number per dish of control cells and TAL1 transduced cells are given. (**B**) Overview of counted colonies upon TAL1 overexpression. G: granulocyte colony, M: monocyte colony, GM: granulocyte-monocyte colony, BFU-E: burst forming unit-erythroid, CFU-E: colony forming unit-erythroid. Colony numbers are given as percent. (**C**) The number of monocytic colonies per dish upon TAL1 overexpression is given. (**D**) The number of erythroid colonies per dish upon TAL1 overexpression is given. (**E**–**K**) Influence of miR-17-92 on differentiation. CD34^+^ cells were transduced with miR-17-92 and the empty vector LegoiG2 (control), respectively. GFP^+^ cells were plated out on methylcellulose and colonies were counted 10–14 days later. (**E**) Total colony number per dish of control cells and miR-17-92 transduced cells are given. (**F**) Overview of counted colonies upon miR-17-92 overexpression. G: granulocyte colony, M: monocyte colony, GM: granulocyte-monocyte colony, BFU-E burst forming unit-erythroid, CFU-E colony forming unit-erythroid. Colony numbers are given as percent. (**G**) The number of monocytic colonies per dish upon miR-17-92 overexpression is given. (**H**) The number of erythroid colonies per dish upon miR-17-92 overexpression is given. (**I**) TAL1 mRNA expression level of all colonies on a plate. (**J**) GYPA mRNA expression level of all colonies on a plate. (**K**) CD71 mRNA expression level of all colonies of one plate. (**I**,**J**) The colonies were resuspended in PBS. Relative mRNA expression was determined by qRT-PCR with specific primer pairs. Values give the relative expression levels compared to GAPDH (percent GAPDH expression). All qRT-PCR experiments were performed at least three times. CFU Assays were performed with cells from three different donors. Data from one representative donor are shown error bars show the standard deviation from six values. The p-values were calculated using Student’s t-test. *P < 0.05; **P < 0.01 (**L**) Schematic illustration of the regulatory loop between miR-17-92 and TAL1 and the influence on its interaction partners E47, HEB and LDB1. miR-17-92 reduces TAL1 protein abundance. This has an influence on protein stability of TAL1 interaction partners and dimer formation. Decreased TAL1 expression would increase E47/E47 homodimer formation compared to less activating TAL1/E47 heterodimer formation (indicated by + /−). Distinct dimer formation would alter gene expression and the biological outcome.

TAL1 plays a prominent role in hematopoietic stem cells and during lineage decision at the megakaryocytic/erythroid branching. The TAL1 complex with E47 plays a role in the activation of many erythroid genes^27,43,48–50^. TAL1 plays a crucial role during differentiation towards erythrocytes and miR-17-92 is expressed in erythroid precursor cells (Fig. S8)^51^. Because miR-17-92 decreases TAL1 activity, we expected the microRNA cluster to negatively influence differentiation processes in which TAL1 is positively involved, such as erythropoiesis. We chose a CFU-assay with primary human CD34^+^ progenitor cells as test system and initially validated the role of TAL1 on erythropoiesis in this system. TAL1 was over expressed in CD34 + cells by lentiviral transduction. GFP-positive transduced cells were plated on methylcellulose. TAL1 expression did not significantly alter the total colony number (Fig. 7A). Similarly, the number of granulocytic colonies remained unchanged (Fig. 7B). Also, macrophage colony number did not change significantly upon TAL1 expression (Fig. 7B,C). As expected, the number of erythroid colonies was increased upon TAL1 expression (Fig. 7D), which confirms the positive role of TAL1 in erythropoiesis.

Subsequently, we tested if miR-17-92 would impact erythropoiesis in the CFU-assay. For this, we expressed miR-17-92 in primary human CD34^+^ cells. GFP-positive transduced cells were plated on methylcellulose. Expression of miR-17-92 did not alter total colony numbers compared to the empty vector (Fig. 7E). Similarly, the number of granulocytic colonies remained unchanged (Fig. 7F). There was a tendency towards more macrophage colonies upon miR-17-92 expression (Fig. 7F,G). The number of erythroid colonies decreased upon transduction with miR-17-92 (Fig. 7H). Upon resuspension of the colonies a reduction of TAL1 (Fig. 7I) and the erythroid marker GYPA (Fig. 7J) and CD71 (Fig. 7K) was detected at the mRNA level in the miR-17-92 transduced cells. To determine the specific role of miR-19 on erythroid differentiation, we performed a CFU-assay with the miR-19 double mutant (see Fig. S4). However, the results of this assay remained inconclusive, probably due to donor to donor variation (Fig. S9).

Our results establish a role of miR-17-92 in erythropoiesis, which could be mediated in part through an influence on TAL1 (Fig. 7L).

## Discussion

A regulatory network of transcription factors, epigenetic modulators and microRNAs establishes and maintains cell type specific gene expression programs. Here, we describe a regulatory loop between miR-17-92 and TAL1. This loop indirectly alters the stability of members of the TAL1 gene regulatory complex.

### Regulation of the TAL1 complex by miR-17-92

We found that overexpression of miR-17-92 reduced TAL1 protein abundance. Interestingly, the amount of the TAL1 heterodimerization partners E47 and HEB was also diminished. However, only evidence for direct activity of miR-17-92 towards TAL1 was collected.

Our data showed that loss of TAL1 expression reduced E47 and HEB at the post-translational level. We demonstrated that TAL1 abundance influences protein stability of E47 and HEB. In particular reduced TAL1 protein amount diminished E47 and HEB stability. These data are in line with the observation that regulation of protein stability is a critical factor for regulation of the TAL1 transcriptional complex. Transcriptional regulation by TAL1 relies on the formation of a multifactorial complex, which contains E47, HEB, LDB1, LMO1/2 and the GATA transcription factors^27,43,52,53^. Notably, interaction with TAL1 reduces proteasomal degradation of LMO2^53,54^. Also, downregulation of LDB1 protein expression by degradation, influences the stoichiometry of associated complexes^55,56^. In light of these results, our observation that TAL1 interaction with E47 stabilizes its heterodimerization partner, supports the notion, that regulation of protein stability is a prominent mechanism to regulate the activity of the TAL1 complex.

### TAL1 regulates miR-17-92 transcription

We detected TAL1 and E47 binding to the promotor of the *MIR17HG* gene, which harbors the miR-17-92 cluster. Chromatin immunoprecipitation revealed binding of TAL1 and E47 at an E-box close to the transcriptional start site in Jurkat, K562 and primary human CD34^+^ progenitor cells. This E-box had been previously described as a binding site for the proto-oncogene c-MYC^47^. Because MYC and TAL1 might bind to the same E-box sequence in the *MIR17HG* promoter a functional cross-influence could be envisioned.

The activity of TAL1 on target genes is dependent on the cofactors it is associated with. TAL1 is able to activate the erythroid gene GYPA in a complex with GATA1, E47, LMO1 and p300/CBP^43^. In other settings, TAL1 is associated with corepressor proteins such as Sin3a and HDAC1^31^. Also, heterodimerization of TAL1 is critical for its function. In particular, LMO2 interaction increases heterodimer formation of TAL1/E47 over E47/E47 homodimer formation^54^. Whereas an E47 homodimer is robustly activating target genes, a TAL1/E47 heterodimer is less transcriptionally active^25^ and shows distinct target gene selection^46,49^. This negative impact of TAL1 on E47 homodimer formation is implicated in cases of T-cell leukemia, where TAL1 is aberrantly expressed^57,58^. In line with these ideas, we observed that the miR-17-92 promoter is activated by E47 and this activation is reduced by TAL1. In line with this notion knockdown of TAL1 increases endogenous miR-17-92 expression in Jurkat and K562 cells. These data indicate that TAL1 reduces activation of miR-17-92 expression partly through interfering with the activity of the E47/E47 homodimer (Fig. 7L).

miR-17-92 probably plays a role during erythroid differentiation as it is expressed in MEPs and early erythroid cells (Fig. S8)^51^. Here, it may be involved in a regulatory loop with the TAL1 complex to keep TAL1/E47 activity at physiological levels. Additionally, it may also act directly on unidentified targets, which play a role in erythropoiesis. In T-cells TAL1 normally is not expressed, except in cases of oncogenic activation, such as in Jurkat cells. However, according to our data, the regulatory loop between TAL1 and miR-17-92 is functional in Jurkat cells. TAL1 and miR-17-92 are also expressed in immature CD34 + progenitor cells and TAL1/E47 are bound to the miR-17-92 promoter in CD34 cells (Fig. 6). Thus, we speculate that presence of TAL1 with miR-17-92 could be representative of an immature T-cell program. If aberrant expression of TAL1 or miR-17-92 could be cause or consequence of a progenitor like state of leukemic cells in T-cells warrants further investigation in primary T-cell material.

### TAL1 and miR-17-92 in differentiation

Consistent evidence points to a role of miR-17-92 in proliferation and differentiation. In hematopoiesis miR-17-92 expands multipotent progenitor cells^10,59^. Furthermore, miR-17-92 is implicated in development of B-cells and T-cells^7,8,60–62^. miR-17-92 expression is connected to several human cancers and regulates expression of genes involved in cell death, cell cycle regulation and proliferation (reviewed in^6^). Several transcription factors with a role in differentiation and proliferation control, have been found to transcriptionally activate miR-17-92, such as RUNX1, EGR2, p53, FLI1 and MYC^47,63–65^ (also see^66^). Transcriptional activation of the miR-17-92 cluster by the transcription factor MYB is implicated in the growth-promoting effects of MYB in Ph-positive leukemia cells^67^. This direct activation of miR-17-92 by MYB was also observed in K562 cells. Thus, it is reasonable that a direct or indirect functional interaction of MYB and TAL1 in regulation of miR-17-92 takes place. Whereas miR-17-92 is expressed in hematopoietic stem cells and undifferentiated cells it is downregulated during differentiation. In myeloid cells this downregulation is triggered by the transcription factors PU.1 and EGR2^64^. Furthermore, p53 and C/EBPß suppress *MIR17HG*^68,69^. The finding that distinct transcription factors are involved in positive and negative regulation of miR-17-92, highlight the fact that regulatory networks of transcription factors and microRNAs are involved in normal hematopoiesis and are disturbed in leukemia.

TAL1 is at the center of hematopoietic transcription factor networks^14,70,71^. Thus, the relationship between TAL1 and miR-17-92 could play a role during TAL1 dependent processes such as erythroid differentiation^72^. In accordance with our results that miR-17-92 inhibits TAL1 expression we found reduced erythroid differentiation in miR-17-92 overexpressing CD34^+^ cells. Deletion of miR-17-92 revealed reduced competitiveness of all hematopoietic progenitor subsets, assumable through an effect on apoptosis^73^. However, we observed no growth advantage of miR-17-92 overexpression in our system. We speculate that the balance of miR-17-92 and TAL1 is important for the cell type specific biological outcome of their interaction (Fig. 7L).

Taken together, we identified a regulatory loop, in which TAL1 expression is repressed by miR-17-92 (Fig. 7L). Reduction of TAL1 protein levels due to miR-17-92 activity indirectly diminishes abundance of TAL1 heterodimerization partners. Because TAL1/E47 heterodimers have lower activating activity than E47/E47 homodimers, an influence of miR-17-92 on the complex formation could be critical for cell fate decision, differentiation and proliferation. Thus, we envision that deregulation of the TAL1/ miR-17-92 axis could be mechanistically important for leukemia/lymphoma.

## Supplementary Information

Supplementary Information

## Data Availability

Proteomics data are available via ProteomeXchange with identifier PXD018052.
