# Supplementary material for: The transcription factor TAL1 and miR-17-92 create a regulatory loop in hematopoiesis
Source: Sci Rep. 2020 Dec 8;10:21438. doi: 10.1038/s41598-020-78629-z (PMC7722897; doi:10.1038/s41598-020-78629-z)
Supplement: Supplementary file 1 — Supplementary Information [file 41598_2020_78629_MOESM1_ESM.pdf]

The transcription factor TAL1 and miR-17-92 create a regulatory loop in hematopoiesis

Annekarin Meyer[1], Stefanie Herkt[1], Heike Kunze-Schumacher[2], Nicole Kohrs[3], Julia Ringleb[3], Lucas Schneider[1], Olga N. Kuvardina[1], Thomas Oellerich[4,5,6], Björn Häupl[4,5,6], Andreas Krueger[2], Erhard Seifried[1], Halvard Bonig[1,7] and Joern Lausen[1,8*]

1. Institute for Transfusion Medicine and Immunohematology, and German Red Cross Blood Service BaWüHe, Goethe University, Sandhofstraße 1, Frankfurt, 60528 Germany.
2. Institute for Molecular Medicine Goethe University, Theodor-Stern-Kai 7, 60590, Frankfurt, Germany.
3. Institute for Tumor Biology and Experimental Therapy, Georg-Speyer-Haus, Paul-Ehrlich-Strasse 42-44, D-60596 Frankfurt am Main, Germany.
4. Department of Medicine II, Hematology/Oncology, Goethe University, Theodor-Stern-Kai 7, 60590 Frankfurt, Germany.
5. German Cancer Research Center and German Cancer Consortium, Heidelberg, Germany
6. Frankfurt Cancer Institute, Goethe University, Frankfurt, 60596, Germany
7. Department of Medicine, Division of Hematology, University of Washington, Seattle, Washington 98195, USA.
8. Institute of Industrial Genetics, Department of Eukaryotic Genetics, University of Stuttgart, Allmandring 31, 70569 Stuttgart, Germany.

* To whom correspondence should be addressed. Tel: +49 (0) 711-685-66971, Email: joern.lausen@iig.uni-stuttgart.de, Jörn Lausen, University of Stuttgart, Department of Eukaryotic Genetics, Institute of Industrial Genetics, Allmandring 31, 70569 Stuttgart, Germany

Supplementary Data

Supplementary Figure 1

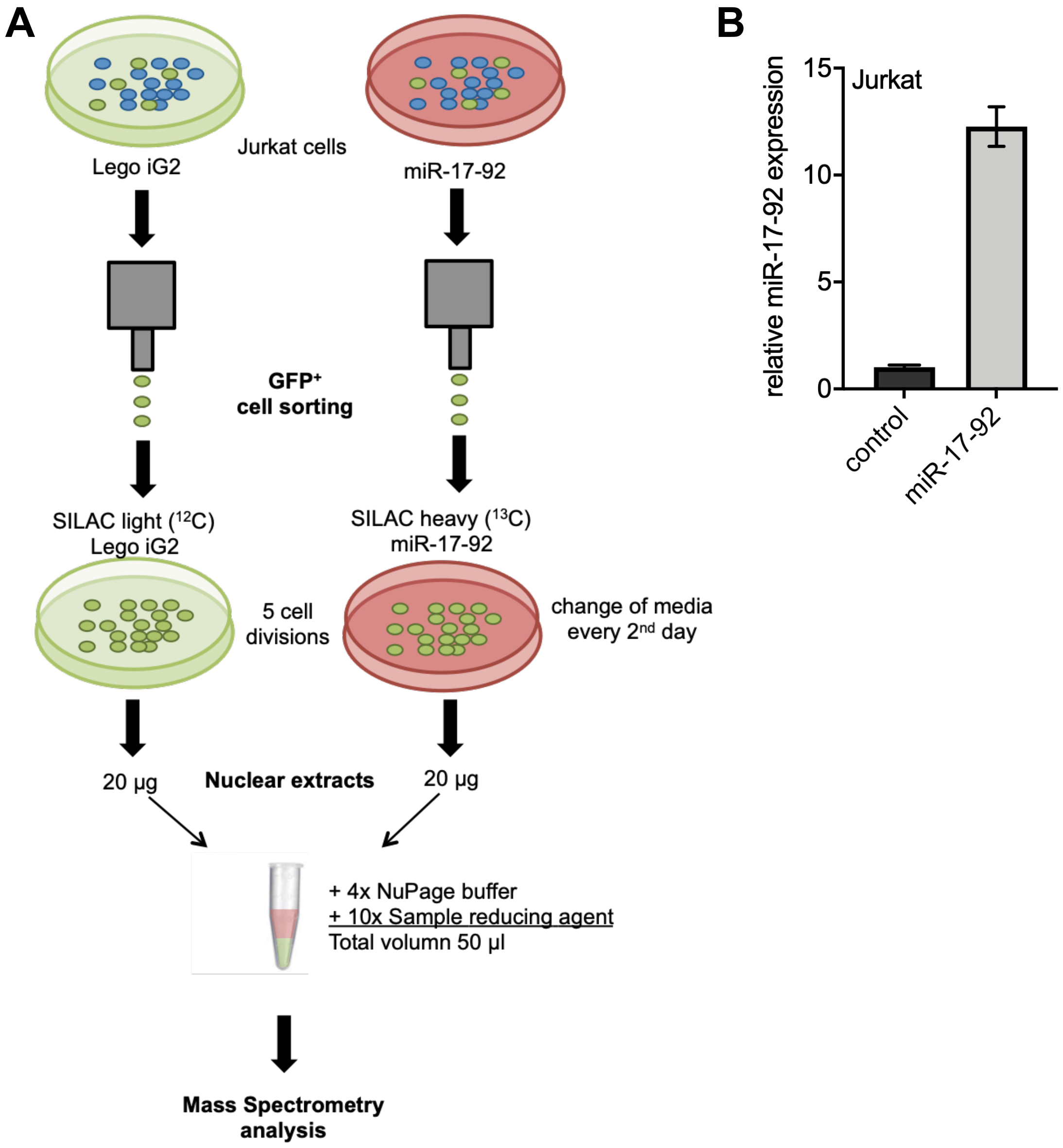

Supplementary Figure 1. A. Schematic representation of the experimental strategy. Jurkat cells were transduced with empty vector or miR-17-92 expression vector. Transduced cells were enriched by sorting with the help of coexpressed GFP. Thereafter the cells were incubated in SILAC medium for five cell devisions and nuclear extracts were prepared. The extracts were mixed in a one to one ratio and analysed by mass spectrometry. **B.** The expression level of miR-17-92 after transduction and sorting was about twelve fold over endogenous expression.

Supplementary Figure 2

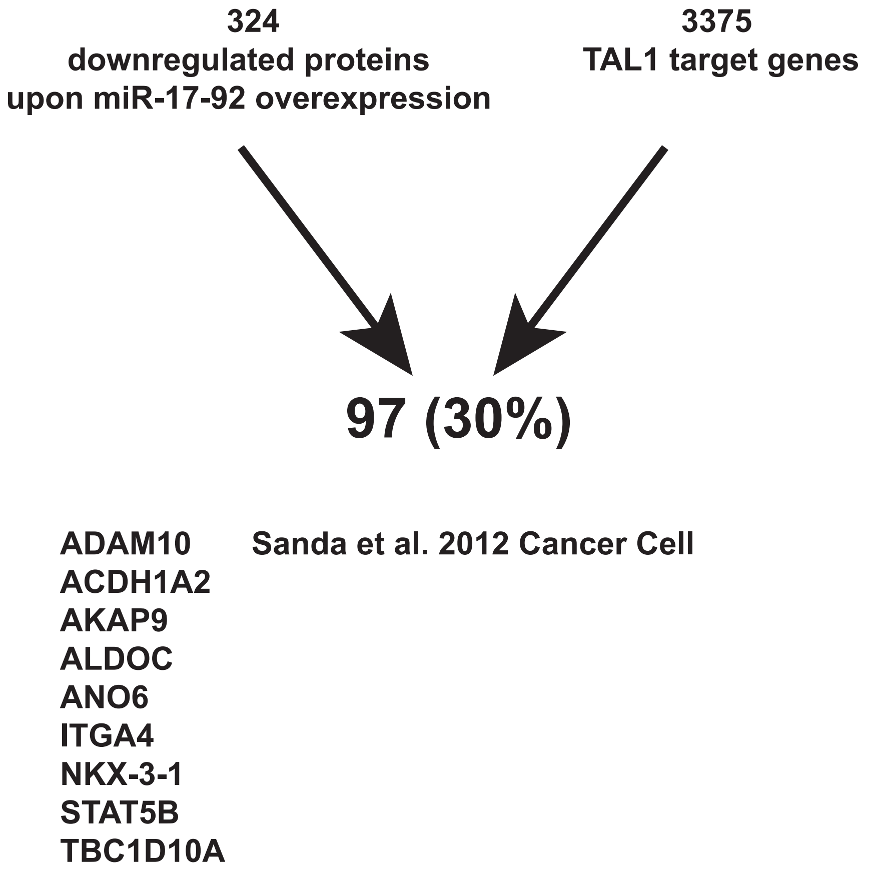

Supplementary Figure 2. The list of 324 proteins, which are downregulated upon miR-17-92 overexpression was compared with TAL1 target genes using the ChEA-database of TAL1 ChIP data. 97 genes displayed TAL1 binding. Furthermore, a subset of miR-17-92 target genes was found in the T-cell context as TAL1 target genes [46].

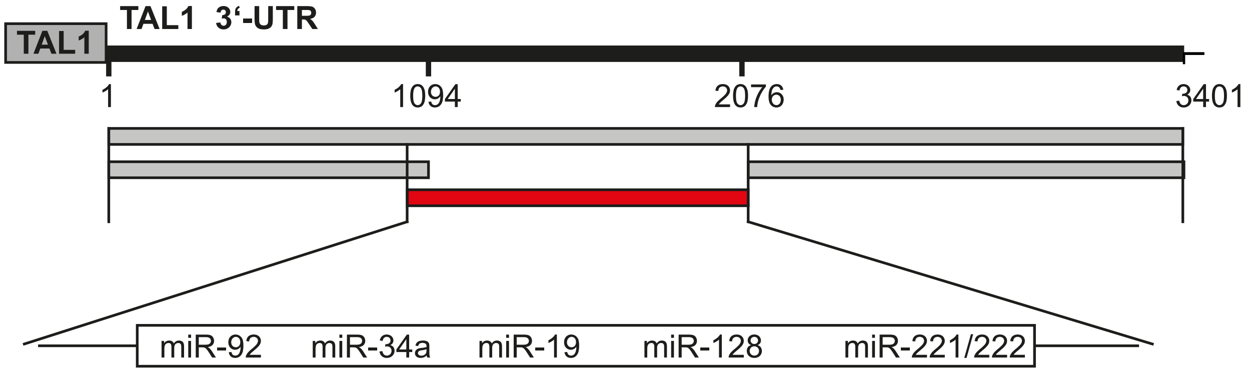

Supplementary Figure 3. Schematic representation of the TAL1 3'-UTR. MicroRNAs of the miR-17-92 cluster are marked. Furthermore, miR-34, miR-128 and miR-221/222 are shown, which have been identified by TargetScan analysis [74].

Supplementary Figure 4

A

miR-17-92 cluster with miR-19a/b sequences:

CTAAATGGACCTCATATCTTTGAGATAATTAAACTAATTTTTTCTTCCCCATTAGGGATTAT
GCTGAATTTGTATGGTTTATAGTTGTTAGAGTTTGAGGTGTTAATTCTAATTATCTATTTCA
AATTTAGCAGGAAAAAAGAGAACATCACCTTGTAAAACTGAAGATTGTGACCAGTCAGAATA
ATGTCAAAGTGCTTACAGTGCAGGTAGTGATATGTGCATCTACTGCAGTGAAGGCACTTGTA
GCATTATGGTGACAGCTGCCTCGGGAAGCCAAGTTGGGCTTTAAAGTGCAGGGCCTGCTGAT
GTTGAGTGCTTTTTGTTCTAAGGTGCATCTAGTGCAGATAGTGAAGTAGATTAGCATCTACT
GCCCTAAGTGCTCCTTCTGGCATAAGAAGTTATGTATTCATCCAATAATTCAAGCCAAGCAA
GTATATAGGTGTTTTAATAGTTTTTGTTTGCAGTCCTCTGTTAGTTTTGCATAGTTGCACTA
CAAGAAGAATGTAGT**TGTGCAAATC**T**ATGCAAAACTGA**TGGTGGCCTGCTATTTCCTTCAAA
TGAATGATTTTTACTAATTTTGTGTACTTTTATTGTGTCGATGTAGAATCTGCCTGGTCTAT
CTGATGTGACAGCTTCTGTAGCACTAAAGTGCTTATAGTGCAGGTAGTGTTTAGTTATCTAC
TGCATTATGAGCACTTAAAGTACTGCTAGCTGTAGAACTCCAGCTTCGGCCTGTCGCCCAAT
CAAACTGTCCTGTTACTGAACACTGTTCTATGGTTAGTTTTGCAGGTTTGCATCCAGCTGTG
TGATATTCTGC**TGTGCAAATC**C**ATGCAAAACTGA**CTGTGGTAGTGAAAAGTCTGTAGAAAAG
TAAGGGAAACTCAAACCCCTTTCTACACAGGTTGGGATCGGTTGCAATGCTGTGTTTCTGTA
TGGTATTGCACTTGTCCCGG*CCTGTTGAGTTTGGTGGGG*ATTGTGACCAGAAGATTTTGAAA
ATTAAATATTACTGAAGATTTCGACTTCCACTGTTAAATGTACAAGATACATGAAATATTAA
AGAAAATGTGTAACTTTTTGTGTAAATACATCTTGTCTTGTTTTC

B

Site directed mutagenesis:

miR-19a mutation: **GTGCAA** → **GTAATA**
miR–19b mutation: **GTGCAA** → **GACCAT**

Supplementary Figure 4. Depiction of the miR-17-92 sequence. **A.** Presentation of the precise location of miR-19a and miR-19b within the miR-17-92 cluster. Underlined are the unprocessed precursor miRNAs of miR-19a (upper sequence) and miR-19b (lower sequence). Shown in bold are the mature miRNAs. The six red nucleotides demonstrate the seed region. The green nucleotide indicates the only difference between miR-19a and miR-19b. **B.** Illustration of the mutated seed region. The exchanged bases are underlined.

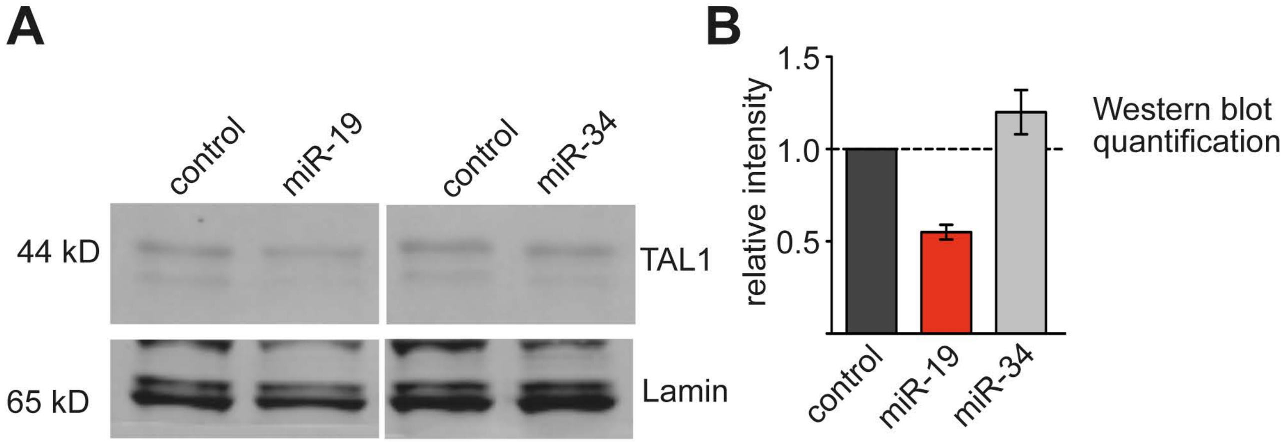

Supplementary Figure 5. A. Western blot analysis of TAL1 expression upon miR-19 and miR-34 over-expression in K562 cells. Left side: TAL1 amount upon overexpression of miR-19. Right side: TAL1 amount upon overexpression of miR-34. An antibody against Lamin was used as loading control. **B.** Quantification of the western blot analysis (n=3). Error bars represent the standart deviation of three independent experiments .

The full blot is shown below.

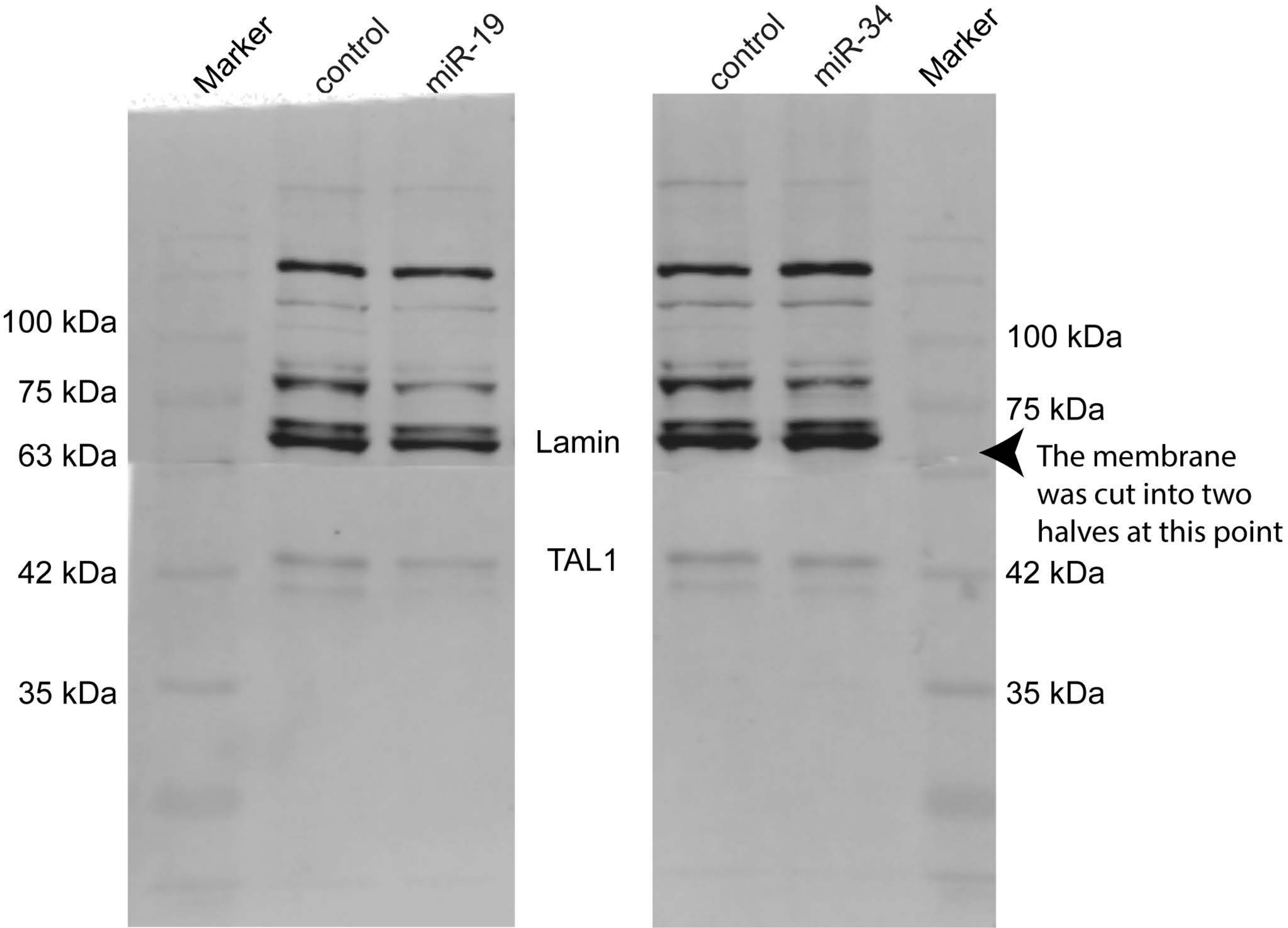

Supplementary Figure 6

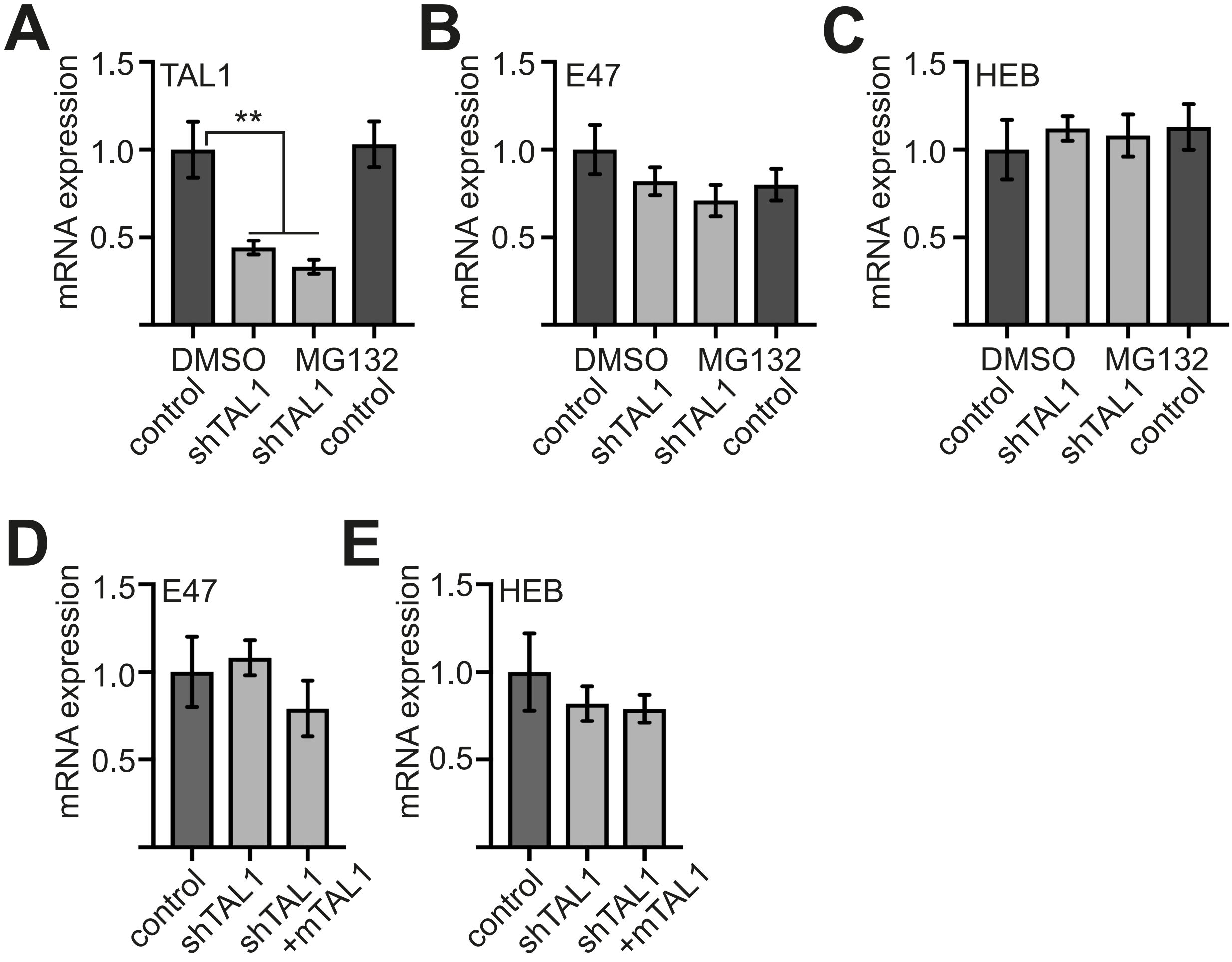

Supplementary Figure 6.
Figure 6A-D are related to Figure 4C in the manuscript. **A.** TAL1 mRNA expression upon knockdown of TAL1 and treatment with the proteasome inhibitor MG132. **B.** E47 mRNA expression upon knockdown of TAL1 and treatment with the proteasome inhibitor MG132. **C.** HEB mRNA expression upon knockdown of TAL1 and treatment with the proteasome inhibitor MG132. Figure 6D-E are related to Figure 4F in the manuscript. **D.** E47 mRNA expression upon knockdown of TAL1 and reintroduction of murine TAL1 (mTal1). **E**. HEB mRNA expression upon knockdown of TAL1 and reintroduction of murine TAL1 (mTal1).
Quantitative rtPCR was performed with specific primer pairs against the given cDNAs. Values were normalized with GAPDH expression and are shown as relative expression compared to cells transduced with an shRNA control vector. Error bars were determined from at least three independent experiments. P-values were calculated using Student's t-test from at least three independent western blots. One representative blot is shown. *P < 0.05; **P < 0.01; ***P < 0.001.

Supplementary Figure 7

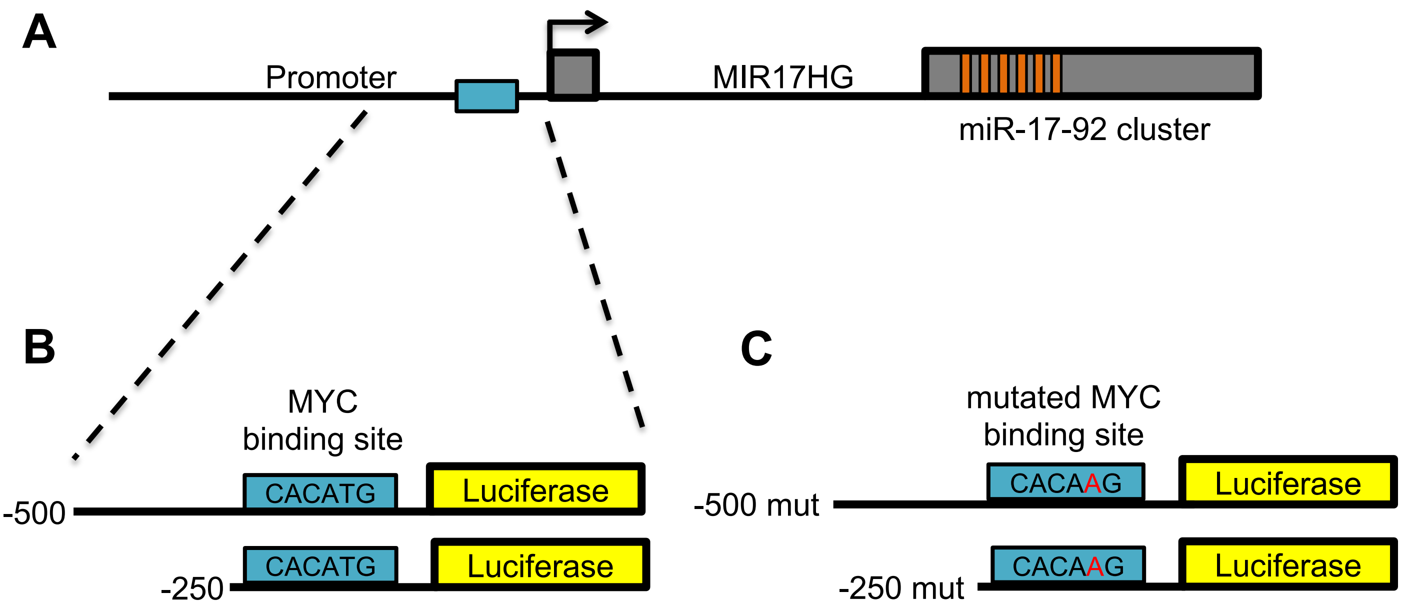

Supplementary Figure 7. A Illustrated is the *MIR17HG* gene with the promoter region, the transcriptional start site, and two non-coding exons (grey boxes), of which the second one contains the miR-17-92 cluster (orange). A known MYC binding site is shown (blue box). **B** Deletion constructs of different length of the promoter region were prepared and cloned in front of a luciferase reporter gene. The MYC binding site is an E-box with the sequence CACATG. **(C)** In order to disrupt the E-box, the sequence was mutated by site directed mutagenesis from CACATG to CACAAG.

Supplementary Figure 8

A

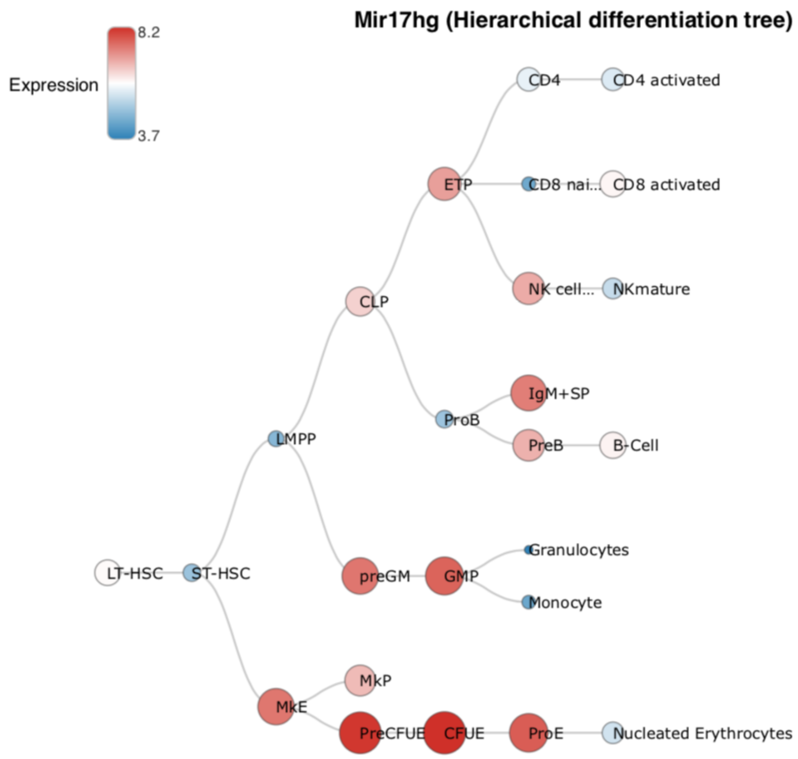

B

Mouse Normal Hematopoiescells are cells are from GSE14833 and GSE6506
Data has been batch corrected

Short	Abbreviation	Phenotype
B-Cell	B-Cell	CD19+ 33D1−
CD4	CD4 T-cells	CD4+ CD25− CD69−
CD4 activated	Activated CD4 positive T-cells	enriched naive T cells with Concanavalin A (1 µg/ml, Sigma) for 8–11 hr followed by sorting for CD25+ and CD69+
CD8 activated	Activated CD8 positive T-cells	enriched naive T cells with Concanavalin A (1 µg/ml, Sigma) for 8–11 hr followed by sorting for CD25+ and CD69+
CD8 naive	Naive CD8 positive T-cells	CD8+ CD25− CD69−
CFUE	Colony-forming unit erythroid cells	c-kit+ Lin− Sca1− CD150- FcgRlow CD105high Ter119-
CLP	Common lymphoid progenitor cells	
ETP	Early T-cell progenitor	
GMP	Granulocyte monocyte progenitors	c-kit+ Lin− Sca1− CD150- FcgRhigh
Granulocytes	Granulocytes	Gr-1+ clone 7/4+ (Cedarlane Labs) CD2− CD5− B220− F4/80− (eBiosciences) ICAM-1− Ter-119−
IgM+SP	Immunoglobulin M positive side population cells	
LMPP	Lymphoid-primed multipotential progenitors	c-kit+ Lin− Sca1+ CD34+ flk2+
LT-HSC	Long term Hematopoietic stem cell	c-kit+ Lin− Sca1+ CD34− flk2−
MkE	Megakaryocyte erythroid precursors	
MkP	Megakaryocyte precursor	
Monocyte	Monocytes	Mac-1+
NK cells	CD56+ natural killer cells	CD56+
NKmature	Mature natural killer cells	Nk1.1+ CD3−
Nucleated Erythrocytes	Nucleated Erythrocytes	Ter-119+ CD3− CD4− CD8− Mac-1− Gr-1− B220−
PreB	Pre-B cell	
PreCFUE	Pre-colony-forming unit erythroid cells	c-kit+ Lin− Sca1− CD150+ FcgRlow CD105hign
ProB	Pro-B cell	
ProE	Erythroid progenitor cells	c-kit+ Lin− Sca1− CD150- FcgRlow CD105high CD71+ Ter119+
ST-HSC	Short term Hematopoietic stem cell	c-kit+ Lin− Sca1+ CD34+ flk2−
preGM	pre-granulocyte monocyte	c-kit+ Lin− Sca1− CD150- FcgRlow CD105low

Quick Guide (click to expand)

Supplementary Figure 8. This Figure shows expression data of *MIR17HG* from mouse hematopoietic cells. Data were taken from the bloodspot databank. Shown are screenshots. **A.** Expression data of MIR17HG as a graphical overview. **B.** Definition of the cell types and marker gene expression used for sorting
Taken from: http://servers.binf.ku.dk/bloodspot/ [15]

Supplementary Figure 9

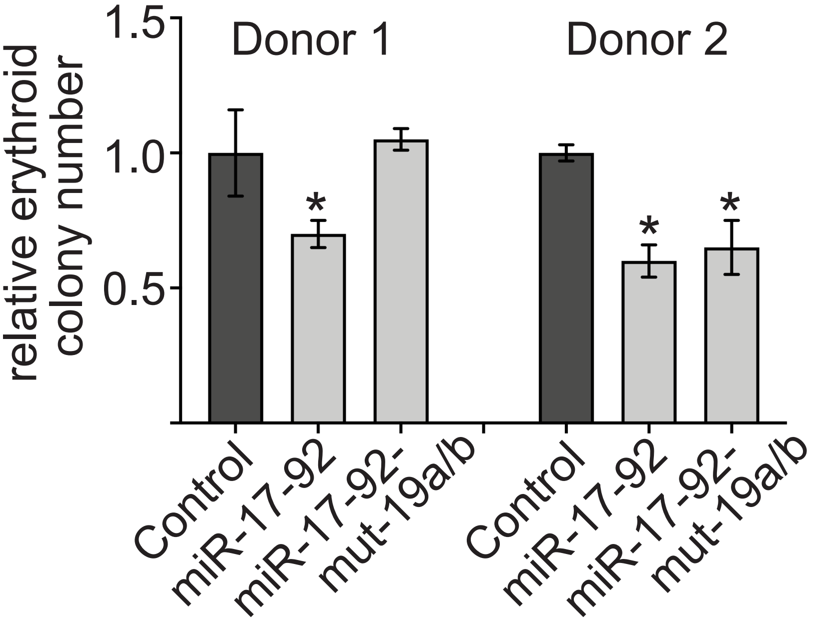

Supplementary Figure 9. Influence of miR-17-92 and miR-17-92-mut-19a/b overexpression on erythroid differentiation of CD34+ cells in a CFU assay. $CD34^+$ cells were transduced with miR-17-92, miR-17-92-mut-19a/b and the empty vector LegoiG2 (control), respectively. GFP^+ cells were plated on methylcellulose and colonies were counted after 10-14 days. The relative number of erythroid colonies per dish upon miR-17-92 and miR-17-92-DM overexpression is given. Values gathered with the control vector were set as one. Data from two representative donors are shown. Error bars show the standard deviation from four values. The p-values were calculated using Student's t-test. *$P < 0.05$

In miR-17-92-mut-19a/b the two miR-19 sites with the miR-17-92 cluster are mutated in their seed sequence. In the first experiment shown on the left side (Donor 1) mutation of miR-19 sites leads to a loss of the effect on erythroid differentiation. In an experiment with $CD34^+$ cells from a different donor (Donor 2), wild type and mutant reduce erythroid colony number to a similar extend. These data indicate, that mutation of the miR-19 sites has a variable effect on erythroid differentiation depending on the cellular and/or genetic background. Furthermore, it is possible that other micro RNAs of the cluster could have an uncharacterized influence on erythroid differentiation.

Related to Figure 1

Supplementary Figure 10

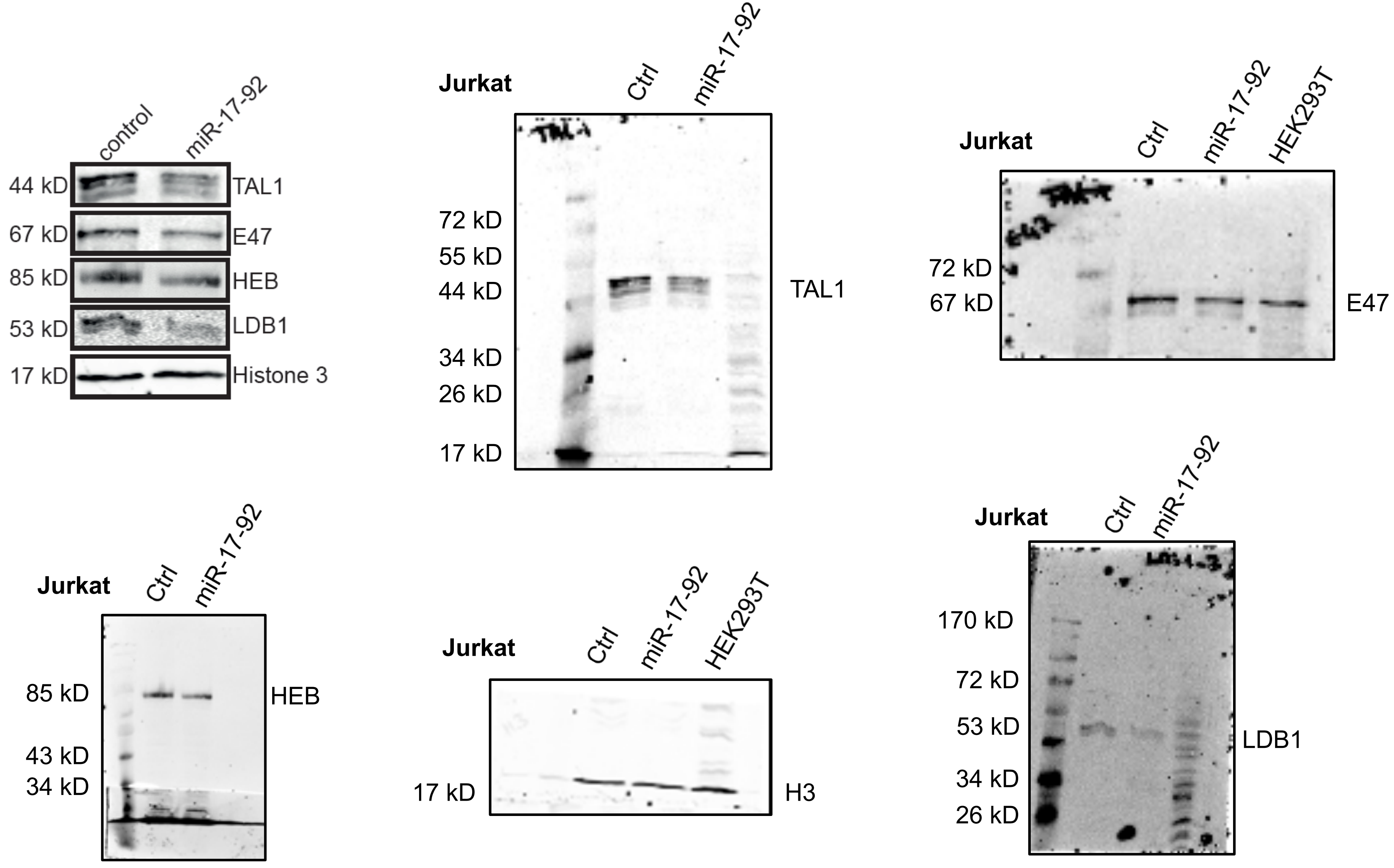

Related to Figure 3

Supplementary Figure 11

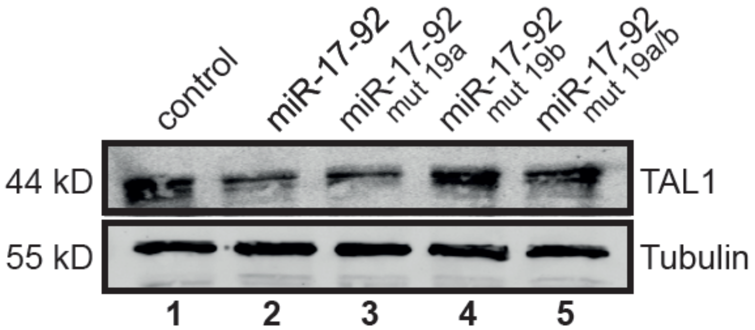

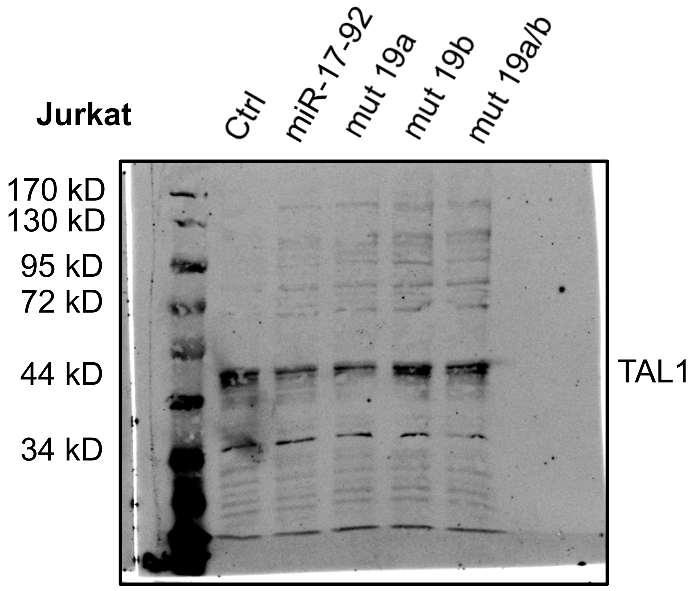

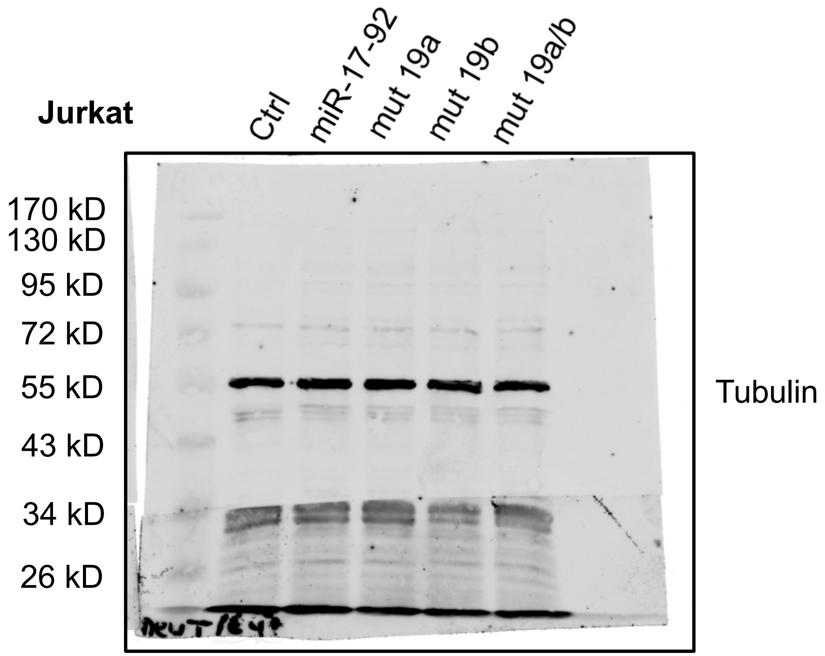

Related to Figure 4 **Supplementary Figure 12**

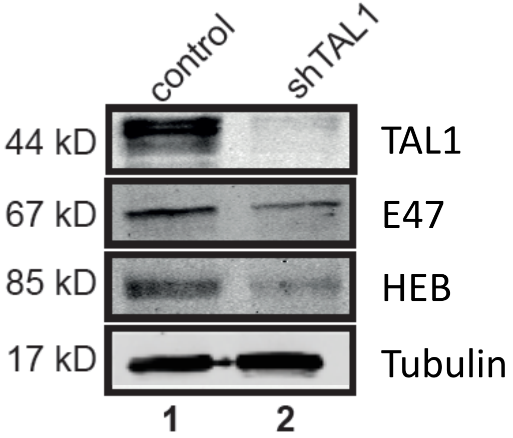

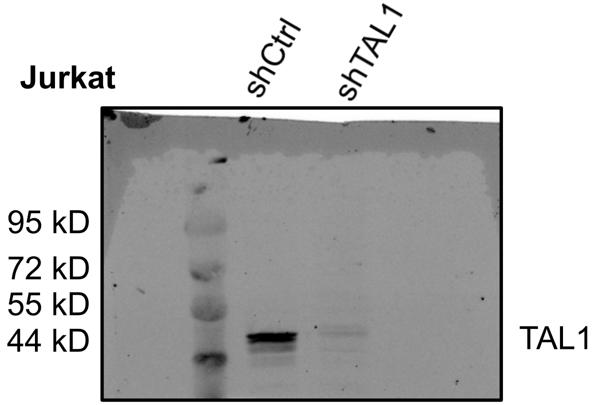

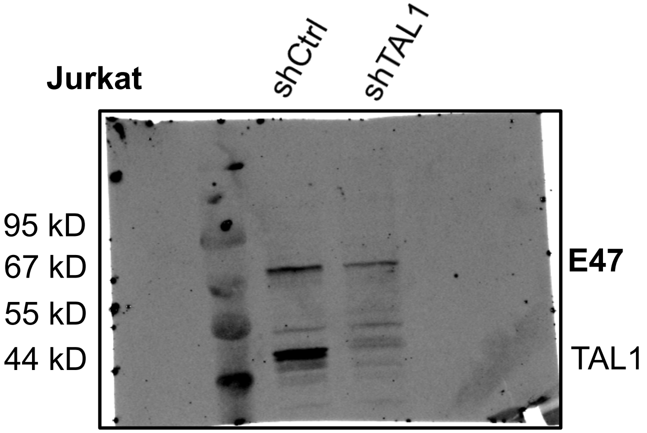

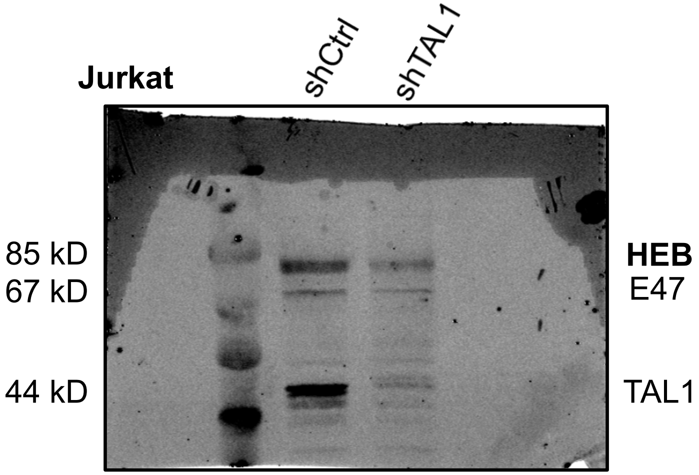

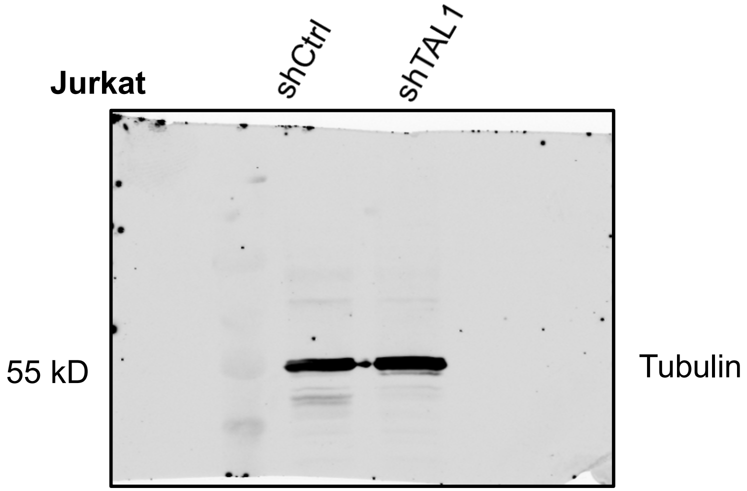

Related to Figure 4

Supplementary Figure 13

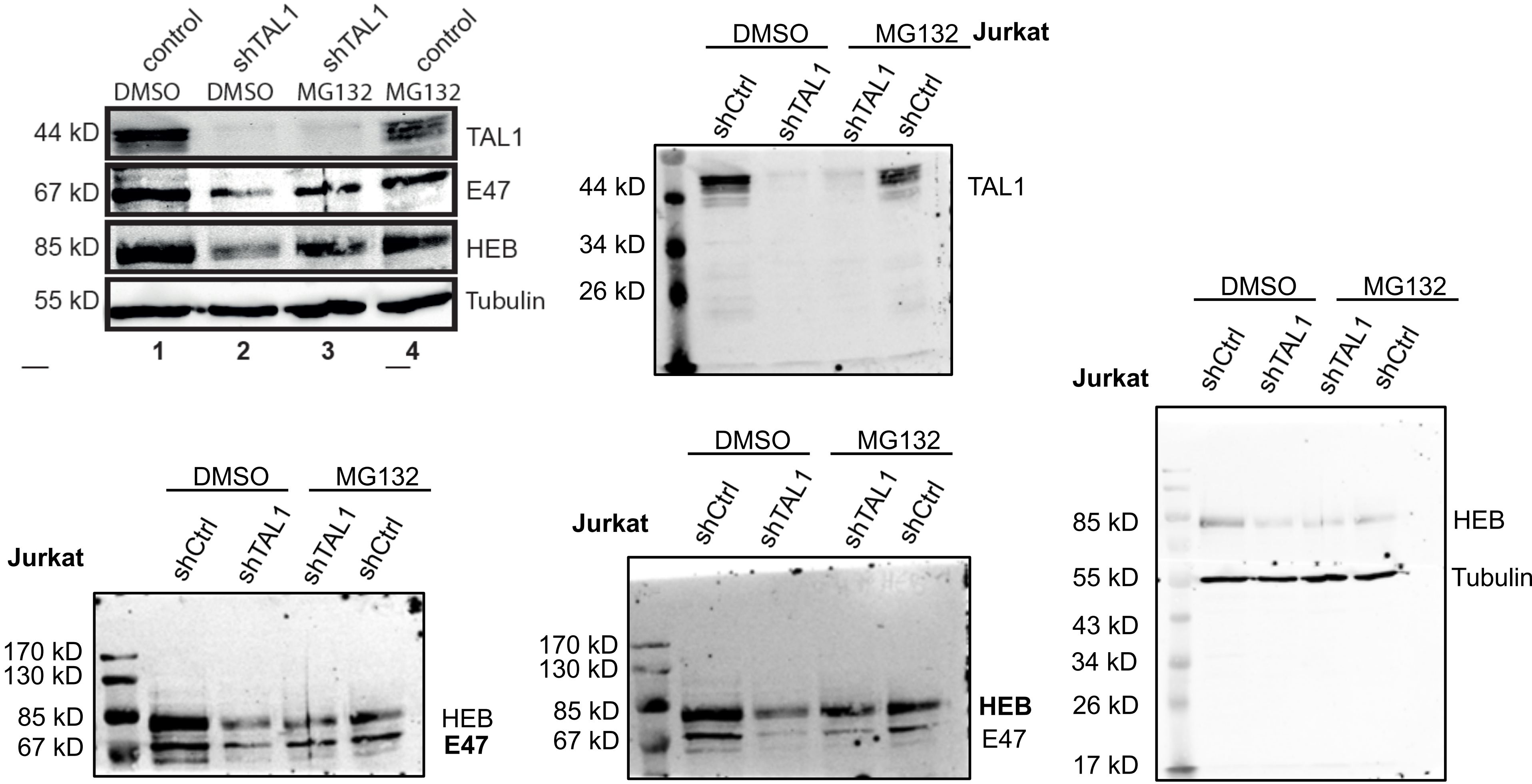

Related to Figure 4

Supplementary Figure 14

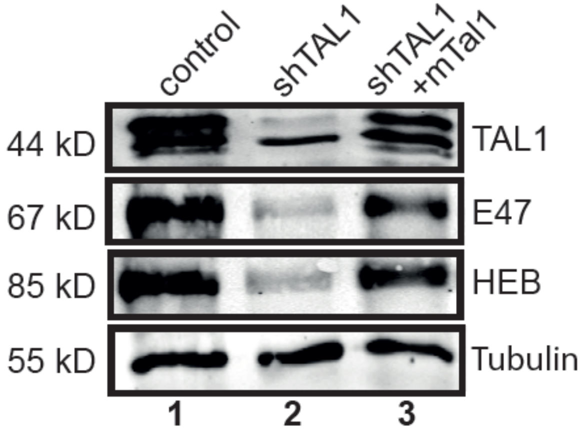

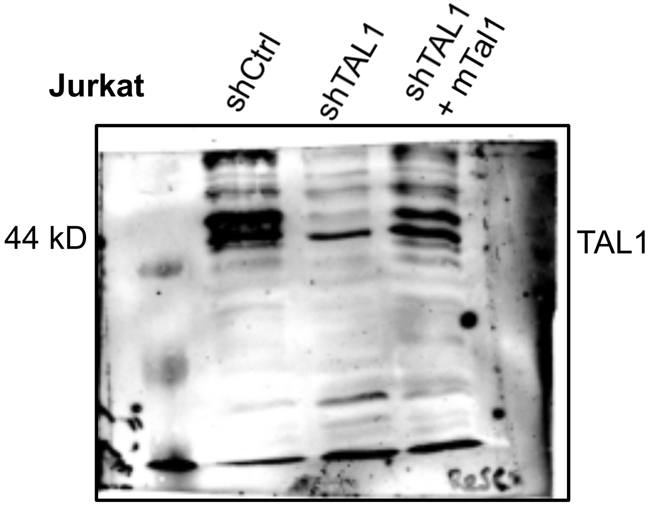

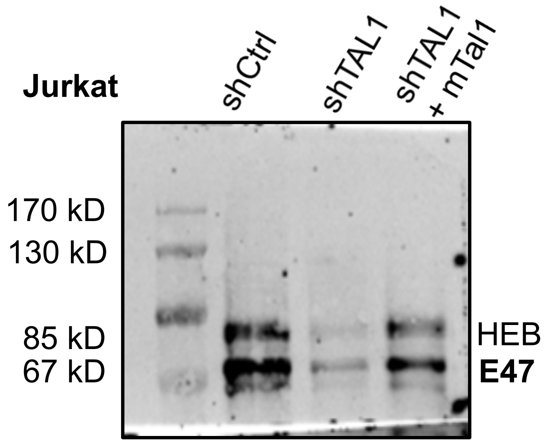

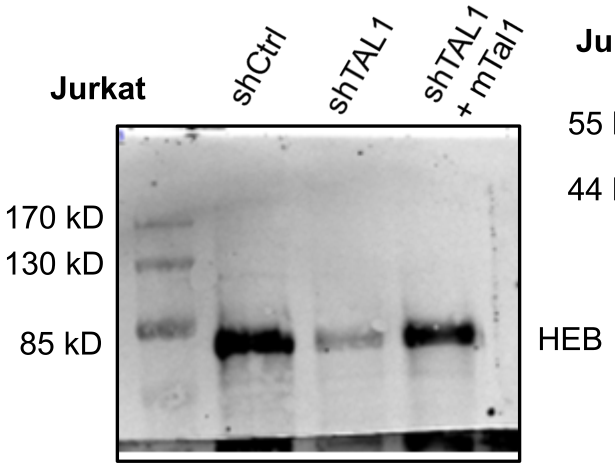

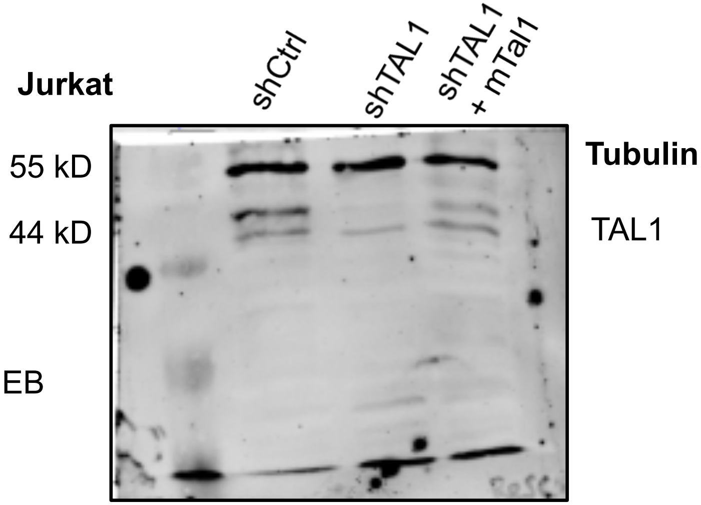

Protein names	Gene names	H/L Ratio
Desmoplakin	DSP;DSP variant protein	0,1932295
Desmoglein-1	DSG1	0,248525
T-cell surface glycoprotein CD4	CD4	0,297545
Keratinocyte proline-rich protein	KPRP	0,304895
Protein EFR3 homolog A	EFR3A	0,318425
Soluble lamin-associated protein of 75 kDa	FAM169A	0,338275
Retinal dehydrogenase 2	ALDH1A2	0,35545
Thy-1 membrane glycoprotein	THY1	0,37713
Mannose-6-phosphate isomerase	MPI	0,381305
Unconventional myosin-VIIb	MYO7B;DKFZp686A08248	0,39272
Phosphorylated adapter RNA export protein	PHAX	0,399325
Zinc finger protein 318	ZNF318	0,4034
DNA nucleotidylexotransferase	DNTT	0,408855
Guanine nucleotide-binding protein G(s) subunit alpha isoforms short;Guanine nucleotide-binding protein G(s) subunit alpha isoforms XLas	GNAS;GSA	0,40949
Eukaryotic translation initiation factor 4E transporter	EIF4ENIF1	0,41228
T-cell receptor beta-1 chain C region;T-cell receptor beta-2 chain C region	TRBC1;TRB;TRBC2;TRBV28;B2M	0,41298
Programmed cell death protein 4	PDCD4	0,421375
Anoctamin;Anoctamin-6	ANO6	0,421375
Flotillin-1	FLOT1	0,431995
Guanine nucleotide-binding protein G(o) subunit alpha	GNAO1	0,438625
Unconventional myosin-Ig;Minor histocompatibility antigen HA-2	MYO1G	0,45025
	FLJ00293	0,451445
Coiled-coil domain-containing protein 88B	CCDC88B	0,455125
Transcription elongation factor B polypeptide 3	TCEB3	0,45695
Unconventional myosin-Ic	MYO1C	0,456955
Uncharacterized protein C3orf17	C3orf17	0,45794
CD109 antigen	CD109	0,46468
Utrophin	UTRN	0,468825
Centromere protein F	CENPF	0,468835
Cyclin-Y	CCNY	0,474635
Raftlin	RFTN1	0,47799
Lactadherin;Lactadherin short form;Medin	MFGE8	0,480235
Fatty acid desaturase 2	FADS2	0,483245
Krueppel-like factor 13	KLF13	0,488285
Fatty acid desaturase 1	FADS1	0,48902
Ketimine reductase mu-crystallin	CRYM	0,490155
Unconventional myosin-Ib	MYO1B	0,49108
Probable ATP-dependent RNA helicase YTHDC2	YTHDC2	0,49108
Leucine-rich repeat flightless-interacting protein 1	LRRFIP1	0,493315
Squalene synthase	FDFT1	0,49513
Helicase ARIP4	RAD54L2	0,49803
Headcase protein homolog	HECA	0,50025
Transcription factor E2-alpha	TCF3	0,5006
Phosphatidylinositol 4-kinase alpha	PIK4CA variant protein;PI4KA;hCG_1999854	0,501475
Lysine-specific demethylase 3B	KDM3B;JMJD1B	0,5038
Arf-GAP with Rho-GAP domain, ANK repeat and PH domain-containing protein 1	ARAP1	0,5043
Lon protease homolog 2, peroxisomal	LONP2	0,51203
Ribonucleoside-diphosphate reductase;Ribonucleoside-diphosphate reductase large subunit	RRM1	0,51418
CD59 glycoprotein	CD59	0,518135
Neogenin	NEO1	0,51942
AT-rich interactive domain-containing protein 4B	ARID4B	0,51945
Splicing factor, arginine/serine-rich 19	SR-A1;SCAF1	0,521255
Regulator of G-protein signaling 19	RGS19	0,52164
Ras-related protein Rap-2b	DKFZp547A0616;RAP2B	0,522325
O-acetyl-ADP-ribose deacetylase MACROD1	MACROD1	0,52366
Signal transducer and activator of transcription;Signal transducer and activator of transcription 5B	STAT5B	0,524355
Zinc finger C3H1 domain-containing protein	ZFC3H1	0,52457
F-box only protein 22	FBXO22	0,525105
Tyrosine-protein kinase ITK/TSK	ITK	0,52659
Rho guanine nucleotide exchange factor 18	ARHGEF18	0,527425
Phosphofurin acidic cluster sorting protein 1	PACS1	0,528115
Copper chaperone for superoxide dismutase;Superoxide dismutase [Cu-Zn]	CCS	0,52924
Golgi-associated plant pathogenesis-related protein 1	GLIPR2;C9orf19	0,53093
RNA-binding protein 33	RBM33	0,53127
Bone marrow stromal antigen 2	BST2	0,533065
Condensin complex subunit 2	NCAPH	0,53563
Tyrosine-protein kinase Lck;Non-specific protein-tyrosine kinase	LCK	0,536265
Guanine nucleotide-binding protein G(i) subunit alpha-2;Guanine nucleotide-binding protein G(i) subunit alpha-1	GNAI2;WUGSC:H_LUCA16.1;GNAI1	0,53671
Guanine nucleotide-binding protein G(I)/G(S)/G(T) subunit beta-1	GNB1	0,53718
SNW domain-containing protein 1	SNW1	0,54043
Transcriptional activator Myb	MYB/NFIB fusion;MYB;c-myb	0,542315
Unconventional myosin-Id	MYO1D;DKFZp686A01173	0,54309
WD repeat domain-containing protein 83	WDR83	0,543195
mRNA-decapping enzyme 1B	DCP1B	0,543995
Protein XRP2	RP2	0,5463
Serine/threonine-protein kinase WNK1	WNK1	0,550865
Protein regulator of cytokinesis 1	PRC1	0,552835

Protein names	Gene names	H/L Ratio
T-cell surface glycoprotein CD3 zeta chain	CD247	0,554235
	EPB41L2	0,55434
Inositol 1,4,5-trisphosphate receptor type 1	ITPR1	0,5545
Transcription factor 7	TCF7	0,555055
ARF GTPase-activating protein GIT1	GIT1	0,55623
Glucocorticoid modulatory element-binding protein 2	GMEB2	0,55687
Leydig cell tumor 10 kDa protein homolog	C19orf53	0,55704
Retinoblastoma-like protein 2	RBL2;DKFZp781K2028	0,557445
TBC1 domain family member 24	TBC1D24	0,55746
Squamous cell carcinoma antigen recognized by T-cells 3	SART3	0,557475
Rho GTPase-activating protein 25	KIAA0053;HEL-S-308;ARHGAP25	0,557615
Lipopolysaccharide-responsive and beige-like anchor protein	LRBA	0,55863
Chromodomain-helicase-DNA-binding protein 1	CHD1	0,562355
TBC1 domain family member 10A	TBC1D10A	0,56289
Synaptosomal-associated protein;Synaptosomal-associated protein 23	SNAP23	0,562935
Guanine nucleotide-binding protein G(k) subunit alpha	GNAI3	0,564325
Hexokinase;Hexokinase-1	HK1	0,564425
Forkhead box protein P4	DKFZp762O213;FOXP4	0,565225
G1/S-specific cyclin-D3	CCND3	0,566665
RING finger protein unkempt homolog	hCG_1776081;UNK	0,56718
Farnesyl pyrophosphate synthase	FDPS	0,56752
Putative Polycomb group protein ASXL1	ASXL1;ASXH1	0,56902
CCR4-NOT transcription complex subunit 3	CNOT3;FLJ00420	0,5716
Protein TANC1	TANC1	0,571685
Calcium/calmodulin-dependent protein kinase type IV	CAMK4	0,572015
Galectin;Galectin-9	LGALS9	0,572015
Chloride intracellular channel protein;Chloride intracellular channel protein 4	CLIC4	0,572905
Mitogen-activated protein kinase kinase kinase kinase;Mitogen-activated protein kinase kinase kinase kinase 2	MAP4K2	0,572985
Guanine nucleotide-binding protein G(I)/G(S)/G(T) subunit beta-2	GNB2	0,574225
General transcription factor IIE subunit 1	GTF2E1	0,574815
Histone H2A deubiquitinase MYSM1	MYSM1	0,575455
Eukaryotic translation initiation factor 4 gamma 3	EIF4G3	0,57596
	DAZAP1/MEF2D fusion	0,577435
Immunoglobulin superfamily member 8	IGSF8	0,5794
NKG2D ligand 2;Retinoic acid early transcript 1G protein	ULBP2;RAET1L;RAET1H;RAET1G	0,580805
DNA replication factor Cdt1	CDT1	0,58132
MAGUK p55 subfamily member 6	MPP6	0,58222
Nuclear receptor corepressor 2	NCOR2	0,583525
Nibrin	NBN	0,58371
Protein Daple	CCDC88C	0,58413
Molybdenum cofactor biosynthesis protein 1;Cyclic pyranopterin monophosphate synthase;Cyclic pyranopterin monophosphate synthase accessory protein	MOCS1	0,58477
Equilibrative nucleoside transporter 1	SLC29A1	0,58491
Mitogen-activated protein kinase;Mitogen-activated protein kinase 9;Mitogen-activated protein kinase 10	MAPK9;MAPK10	0,587885
Ras-related protein Rab-22A	RAB22A	0,5884
Guanine nucleotide-binding protein subunit alpha-14	GNA14	0,58895
Galectin;Galectin-8	LGALS8	0,59042
Interferon regulatory factor 2-binding protein 2	IRF2BP2	0,590945
**Transcription factor 12**	**TCF12**	**0,591475**
Vacuolar protein sorting-associated protein 13A	VPS13A	0,591655
Helicase SKI2W	SKIV2L;SKI2W	0,5917
**Homeobox protein Nkx-3.1**	**NKX3-1**	**0,59245**
G protein-coupled receptor kinase 6	GRK6	0,59412
Guanine nucleotide-binding protein G(q) subunit alpha	GNAQ	0,59426
Aconitate hydratase;Cytoplasmic aconitate hydratase	HEL60;ACO1;IRP1	0,59481
Engulfment and cell motility protein 2	ELMO2	0,59567
E3 ubiquitin-protein ligase RNF168	RNF168	0,59595
Constitutive coactivator of PPAR-gamma-like protein 1	FAM120A	0,597045
RNA-binding protein 26	RBM26	0,59955
PAX-interacting protein 1	PAXIP1	0,599805
Ubiquitin carboxyl-terminal hydrolase 47	USP47	0,600765
Ubiquitin carboxyl-terminal hydrolase 15	USP15	0,601245
Zinc finger CCCH domain-containing protein 4	ZC3H4	0,60155
Syntenin-1	SDCBP	0,60174
Protein strawberry notch homolog 1	SBNO1	0,601875
F-box only protein 7	FBXO7;DKFZp686B08113	0,60192
Protein SCAF11	SCAF11;SFRS2IP	0,602555
MKL/myocardin-like protein 2	MKL2	0,602625
Inactive tyrosine-protein kinase 7	PTK7;DKFZp434L0319	0,603385
RING finger and SPRY domain-containing protein 1	RSPRY1	0,60344
Protein prune homolog	PRUNE	0,603595
FYN-binding protein	FYB	0,604105
Negative elongation factor E	NELFE;RDBP;NELF-E	0,60437
Proto-oncogene tyrosine-protein kinase Src	SRC	0,606475
DnaJ homolog subfamily C member 5	DNAJC5;DKFZp761N1221;FLJ00095	0,606685
Integrin beta-1	ITGB1	0,606855
Intersectin-2	ITSN2	0,608605
Mitogen-activated protein kinase kinase kinase kinase 4	MAP4K4	0,608785

Protein names	Gene names	H/L Ratio
Intercellular adhesion molecule 3	ICAM3;hCG_2033729	0,61071
Double-stranded RNA-binding protein Staufen homolog 2	STAU2;STAU2 variant protein	0,611255
Tetratricopeptide repeat protein 27	TTC27	0,611985
Ras-related protein Rab-23	RAB23	0,612075
Hematological and neurological expressed 1 protein;Hematological and neurological expressed 1 protein, N-terminally processed	HN1	0,61398
Poly(A) polymerase alpha	PAPOLA	0,61462
AP-3 complex subunit delta-1	AP3D1	0,61465
Ubiquitin carboxyl-terminal hydrolase 24	USP24	0,615145
A-kinase anchor protein 2	hCG_28765;AKAP2	0,615995
Integrin alpha-L	ITGAL	0,617175
Coilin	COIL	0,61735
Zinc finger E-box-binding homeobox 1	ZEB1	0,617495
**Myocyte-specific enhancer factor 2D**	**MEF2D variant protein;MEF2D**	**0,61758**
Eukaryotic translation initiation factor 2-alpha kinase 4	EIF2AK4	0,61789
F-box/LRR-repeat protein 8	FBXL8	0,618815
Myotubularin-related protein 1	MTMR1	0,618955
B-cell CLL/lymphoma 9 protein	BCL9	0,61971
Zinc finger BED domain-containing protein 1	hCG_1981838;ZBED1	0,619715
Proteasome maturation protein	POMP	0,62158
Fatty acid synthase;[Acyl-carrier-protein] S-acetyltransferase;[Acyl-carrier-protein] S-malonyltransferase;3-oxoacyl-[acyl-carrier-protein] synthase;3-oxoacyl-[acyl-carrier-protein] reductase;3-hydroxyacyl-[acyl-carrier-protein] dehydratase;Enoyl-[acyl-carrier-protein] reductase;Oleoyl-[acyl-carrier-protein] hydrolase	FASN	0,621665
Male-specific lethal 1 homolog	MSL1;hCG_31740	0,622265
Ras-related protein Rab-4A	RAB4A	0,62315
RNA-binding protein 27	RBM27;POU4F3	0,6238
Myotubularin-related protein 5	SBF1;DKFZp761D0422	0,62391
60S ribosomal protein L34	RPL34	0,62444
Pumilio homolog 1	PUM1	0,626135
Protein SDE2 homolog	SDE2	0,6263
Very low-density lipoprotein receptor	VLDLR	0,626335
Telomeric repeat-binding factor 2-interacting protein 1	TERF2IP	0,62637
WD repeat-containing protein 37	WDR37	0,6268
Niban-like protein 1	C9orf88;FAM129B	0,62874
Pre-mRNA-processing factor 17	CDC40	0,630325
**LIM domain-binding protein 1**	**LDB1**	**0,63037**
Mth938 domain-containing protein	AAMDC	0,630445
Na(+)/H(+) exchange regulatory cofactor NHE-RF2	SLC9A3R2	0,63111
Nucleolar protein 8	NOL8;DKFZp686P12242	0,63139
Nuclear receptor corepressor 1	NCOR1	0,6316
Protein-tyrosine kinase 2-beta	PTK2B	0,631775
Probable ATP-dependent RNA helicase DDX27	DDX27	0,63364
CLIP-associating protein 1	CLASP1	0,634125
Death domain-associated protein 6	DAXX	0,63432
Replication initiator 1	REPIN1	0,63475
1-phosphatidylinositol 3-phosphate 5-kinase	PIKFYVE;HEL37	0,63557
Nischarin	NISCH	0,635575
Neurofibromin;Neurofibromin truncated	NF1	0,635695
Ran-binding protein 9	RANBP9	0,63603
Probable ATP-dependent RNA helicase DDX20	DDX20	0,636345
**T-cell acute lymphocytic leukemia protein 1**	**tal-1;TAL1;SCL**	**0,63754**
Nicotinate phosphoribosyltransferase	NAPRT	0,637675
Pre-mRNA-splicing factor CWC25 homolog	CWC25	0,637875
E3 ubiquitin-protein ligase CBL	CBL	0,637985
tRNA 2-phosphotransferase 1	TRPT1	0,639385
Peptidyl-prolyl cis-trans isomerase;FK506-binding protein 15	FKBP15	0,64004
Diphosphoinositol polyphosphate phosphohydrolase 2	NUDT4	0,641005
SLAIN motif-containing protein 2	hCG_17415;SLAIN2	0,64197
NFATC2-interacting protein	NFATC2IP	0,64425
Mediator of RNA polymerase II transcription subunit 12	MED12;TNRC11	0,646005
Abelson tyrosine-protein kinase 2	ABL2	0,646485
Alpha-ketoglutarate-dependent dioxygenase FTO	FTO	0,647725
RNA polymerase II subunit A C-terminal domain phosphatase	CTDP1	0,647755
Disco-interacting protein 2 homolog B	DIP2B	0,64836
E3 ubiquitin-protein ligase HECTD3	HECTD3	0,64865
TBC1 domain family member 15	TBC1D15	0,648915
Isovaleryl-CoA dehydrogenase, mitochondrial	IVD	0,649325
Anion exchange protein;Sodium bicarbonate cotransporter 3	SLC4A7	0,65124
Interferon regulatory factor 2-binding protein-like	IRF2BPL	0,65127
MKI67 FHA domain-interacting nucleolar phosphoprotein	NIFK	0,6525
DCC-interacting protein 13-alpha	APPL1	0,653995
Ubiquitin carboxyl-terminal hydrolase;Ubiquitin carboxyl-terminal hydrolase BAP1	BAP1	0,65416
Intercellular adhesion molecule 2	ICAM2	0,65482
Ribonuclease 3	RNASEN;DROSHA	0,654835
Putative 60S ribosomal protein L39-like 5;60S ribosomal protein L39	RPL39P5;RPL39	0,65525
Inhibitor of nuclear factor kappa-B kinase subunit beta	IKBKB	0,656525
PR domain zinc finger protein 10	PRDM10	0,6566
2-deoxynucleoside 5-phosphate N-hydrolase 1	DNPH1	0,656835
Carabin	TBC1D10C	0,65752
Tripartite motif-containing protein 65	TRIM65	0,65833

Protein names	Gene names	H/L Ratio
Pleckstrin homology domain-containing family A member 1	PLEKHA1	0,65969
Shootin-1	KIAA1598	0,659815
Alanine--tRNA ligase, mitochondrial	AARS2	0,660235
ARF GTPase-activating protein GIT2	GIT2	0,660345
Ubiquitin-associated protein 2	UBAP2;UNQ610	0,66076
Translation initiation factor eIF-2B subunit epsilon	EIF2B5	0,661135
ATR-interacting protein	TREX1;ATRIP	0,662325
Leucine-rich repeat-containing protein 16C	RLTPR	0,662585
Protein LYRIC	MTDH	0,662795
SWI/SNF-related matrix-associated actin-dependent regulator of chromatin subfamily A-like protein 1	SMARCAL1	0,662945
Tubulin epsilon chain	TUBE1	0,663135
Tuberin	TSC2	0,663735
Isobutyryl-CoA dehydrogenase, mitochondrial	ACAD8	0,66423
40S ribosomal protein S27;40S ribosomal protein S27-like	RPS27L;LOC392748	0,664415
Trafficking protein particle complex subunit 9	TRAPPC9	0,666445
NEDD8 ultimate buster 1	NYREN18;NUB1	0,667585
Rootletin	CROCC	0,66798
Calmodulin	CALM3;CALM2;CALM1	0,668435
Transcription factor Dp-2	TFDP2	0,66899
Zinc finger protein 598	ZNF598	0,66962
Mismatch repair endonuclease PMS2	PMS2	0,670915
Golgi-specific brefeldin A-resistance guanine nucleotide exchange factor 1	GBF1	0,670985
Phospholipase D3	PLD3	0,67103
Switch-associated protein 70	SWAP70	0,671425
Regulatory factor X-associated protein	RFXAP	0,67364
Transcription factor BTF3;Transcription factor BTF3 homolog 4	BTF3L4	0,67416
N6-adenosine-methyltransferase subunit METTL14	METTL14	0,674775
Zinc finger and BTB domain-containing protein 7A	ZBTB7A	0,67513
Protein FAM91A1	FAM91A1	0,67521
Serine/threonine-protein kinase tousled-like 1	TLK1	0,675355
Cyclin-dependent kinase 12	CDK12	0,67544
Calcium-transporting ATPase;Plasma membrane calcium-transporting ATPase 4	ATP2B4;DKFZp686M088	0,675805
5-3 exoribonuclease 1	XRN1	0,6766
FLYWCH family member 2	FLYWCH2	0,67757
TNF receptor-associated factor 6	TRAF6	0,677745
Amino acid transporter;Neutral amino acid transporter A	SLC1A4	0,67869
Serine/threonine-protein kinase TAO1	TAOK1	0,678835
E3 ubiquitin-protein ligase UBR2	UBR2	0,67953
Transcription initiation factor TFIID subunit 3	TAF3	0,67956
UBX domain-containing protein 6	UBXN6	0,679755
Cyclin-T1	CCNT1	0,68165
Cytochrome P450 2U1	CYP2U1	0,681765
Activated RNA polymerase II transcriptional coactivator p15	PC4;SUB1	0,68215
CD48 antigen	CD48	0,68215
Formin-binding protein 1-like	FNBP1L	0,68219
Ras-related protein Rab-5B	RAB5B	0,682255
Ras-related protein Ral-A	RALA	0,682575
Guanine nucleotide-binding protein subunit beta-like protein 1	GNB1L	0,682925
Signal transducer and activator of transcription;Signal transducer and activator of transcription 5A	STAT5A	0,68369
Ankyrin repeat and zinc finger domain-containing protein 1	ANKZF1	0,683825
Protein PRRC2C	PRRC2C	0,68417
ADP-ribosylation factor GTPase-activating protein 3	ARFGAP3	0,68448
La-related protein 4B	LARP4B	0,68469
Disks large homolog 1	DLG1	0,68538
Tropomyosin alpha-1 chain	TPM1	0,68539
UBX domain-containing protein 7	UBXN7	0,685775
Non-structural maintenance of chromosomes element 4 homolog A	NSMCE4A	0,68621
Microtubule-associated protein 1A;MAP1A heavy chain;MAP1 light chain LC2	MAP1A	0,686235
GRIP1-associated protein 1	GRIPAP1	0,687445
Lysosome membrane protein 2	SCARB2	0,68754
AP-3 complex subunit beta		0,68792
Conserved oligomeric Golgi complex subunit 1	COG1	0,6909
Protein BRICK1	BRK1	0,69125
DNA-binding protein RFXANK	RFXANK	0,691435
Acyl-coenzyme A oxidase;Peroxisomal acyl-coenzyme A oxidase 1	ACOX1	0,69158
Selenocysteine lyase	SCLY	0,692335
High mobility group protein 20A	HMG20A	0,692475
Max-binding protein MNT	MNT	0,69349
DNA primase large subunit	PRIM2	0,694265
Autophagy-related protein 101	ATG101;C12orf44	0,69489
Bifunctional epoxide hydrolase 2;Cytosolic epoxide hydrolase 2;Lipid-phosphate phosphatase	EPHX2	0,69498
Gamma-enolase;Enolase	ENO2	0,695485
Fructose-bisphosphate aldolase;Fructose-bisphosphate aldolase C	ALDOC	0,69574
Protein Red	CSA2;IK	0,69595
Probable leucine--tRNA ligase, mitochondrial	LARS2	0,696335
Nucleus accumbens-associated protein 1	BTBD14B;NACC1	0,69636
Annexin	ANXA6	0,696445

Protein names	Gene names	H/L Ratio
UPF0769 protein C21orf59	C21orf59	0,69658
GDNF family receptor alpha-1	GFRA1	0,696645
Uncharacterized protein KIAA1143	KIAA1143	0,696985
Probable ATP-dependent RNA helicase DDX60	DDX60	0,697415
Lysine-specific demethylase 4B	KDM4B;JMJD2B	0,69744
Serine/threonine-protein kinase SMG1	SMG1	0,69774
RNA pseudouridylate synthase domain-containing protein 3	RPUSD3	0,698095
1,2-dihydroxy-3-keto-5-methylthiopentene dioxygenase	ADI1	0,69841
Mediator of RNA polymerase II transcription subunit 25	MED25	0,698535
SRSF protein kinase 2;SRSF protein kinase 2 N-terminal;SRSF protein kinase 2 C-terminal	SRPK2	0,698815
E3 ubiquitin-protein ligase TRIM38	TRIM38;DKFZp686F23130	0,69912
DNA repair and recombination protein RAD54-like	RAD54L	0,69918
Lambda-crystallin homolog	HEL30;CRYL1	0,699425
Protein ENL	MLLT1	0,699605
UPF0769 protein C21orf59	C21orf59	0,69658
GDNF family receptor alpha-1	GFRA1	0,696645
Uncharacterized protein KIAA1143	KIAA1143	0,696985
Probable ATP-dependent RNA helicase DDX60	DDX60	0,697415
Lysine-specific demethylase 4B	KDM4B;JMJD2B	0,69744
Serine/threonine-protein kinase SMG1	SMG1	0,69774
RNA pseudouridylate synthase domain-containing protein 3	RPUSD3	0,698095
1,2-dihydroxy-3-keto-5-methylthiopentene dioxygenase	ADI1	0,69841
Mediator of RNA polymerase II transcription subunit 25	MED25	0,698535

Supplementary Table 1: Summary of all 324 SILAC-analysed proteins with an H/L ratio smaller than 0.7. Given are the protein names and gene names and the mean of two analysed replicates which were achieved by the evaluation of the SILAC data.

Table S2: Real-time primer

Gene name	Sequence 5´→3
hTAL1-f<br>hTAL1-r	TCGGCAGCGGGTTCTTTGGG<br>CCATCGCTCCCGGCTGTTGG
mTal1-f<br>mTal1-r	GCTCGCCTCACTAGG<br>CACCCGGTTGTTGTT
GAPDH-f<br>GAPDH-r	TCTTTTGCGTCGCCAGCCGAGC<br>TGACCAGGCGCCCAATACGACC
HEB-f<br>HEB-r	AGGGAATGCTGCTGGAAGCTCA<br>CTGGTACCTGTGAGAGGTGAAGGT
E47-f<br>E47-r	GGGACTCGGAGGCAAGAGCG<br>TCGCCTGACAGGAAGCCAGC
CD71-f<br>CD71-r	AGGACGCGCTAGTGTTCTTC<br>CCAGGCTGAACCGGGTATATG
GpA-f<br>GpA-r	CCCTCCAGAAGAGGAAACCGGAGA<br>GGCACGTCTGTGTCAGGTGAGG
miR-17-92-f<br>miR-17-92-r	TTTGCATCCAGCTGTGTGAT<br>ACCGATCCCAACCTGTGTAG
LDB1-f<br>LDB1-r	ACCTGATGTGATGGTGGTGG<br>TGCGTCAAACTGGGTGTTCT

Table S3: ChIP primer

Gene name	Sequence 5´→3
GAPDH E6-f<br>GAPDH E6-r	GCCAAGGCTGTGGGCAAGGT<br>CCTCCGACGCCTGCTTCACC
GpA prom-f<br>GpA prom-r	CAGGCGCTTAACAACTTGCATCA<br>CATACATCCTGAGATCATGAGCTG
MIR17HG myc-f<br>MIR17HG myc-r	GAGTGGGGCTTGTCCGTATTT<br>CCTCGAAGGACCATGTGGGT
MIR17HG -4000-f<br>MIR17HG -4000-r	GCCCCACTTCTTACCACCTA<br>CCCAGCCCAGCTGTACTTTAC
CD4 enhancer-f<br>CD4 enhancer-r	GGGTCGGTTCTATCTGCTCCA<br>GCTCCAGGACCACTTTTTGCC

Table S4: Primary antibodies

Name	Host species	Mono/polyclonal	Cat-number	manufacturer	dilution
Alpha-Tubulin (DM1A)	mouse	monoclonal	ab7291	Abcam	1:5000
Actin	rabbit	polyclonal	ab1801	Abcam	1:1000
H3	rabbit	polyclonal	ab1791	Abcam	1:1000
Tal1 (BTL73)	mouse	monoclonal	04-123	Millipore	1:500
E47 (G-2)	mouse	monoclonal	sc-133075	Santa Cruz	1:500
HEB/TCF 12 D2C10	rabbit	monoclonal	#11825	Cell Signaling	1:1000
LDB1	mouse	monoclonal	sc-365074	Santa Cruz	1:1000

Table S5: Secondary antibodies

Name	Host species	Cat-number	manufacturer	dilution
anti-rabbit IRDye 800CW	donkey	926-32213	Li-cor	1:15000
anti-mouse IRDye 800CW	donkey	926-32212	Li-cor	1:15000

Table S6: ChIP antibodies

Name	Host species	Mono/polyclonal	Cat-number	manufacturer	concentration
Tal1 (GAT)	rabbit	polyclonal	TA590662-OR	OriGene (BioCat)	4µg
E47 (TCF3/E2A)	rabbit	polyclonal	ab11176	Abcam	4µg
H3	rabbit	polyclonal	ab1791	Abcam	3µg
H3 K9 ac	rabbit	polyclonal	ab10812	Abcam	3µg
